# Unraveling anti-atherosclerosis mechanism of anthocyanins from Xinjiang wild cherry plum (*Prunus divaricata* Ledeb) via network pharmacology and molecular docking

**DOI:** 10.1186/s40643-025-00900-w

**Published:** 2025-06-06

**Authors:** Siyu Li, Juan He, Huiyi Hu, Guang Wang, Juan Tang, Jun Yao, Jing Shen, Xing Li

**Affiliations:** 1 PuAi Medical School, Shaoyang University, Shaoyang, 422000 China; 2 Medical College, Hunan University of Medicine, Huaihua, 418000 China; 3 Anhui Bioengineering School, Hefei, 230031 China; 4 College of Pharmacy, Xinjiang Medical University, Urumqi, 830017 China; 5 Department of Pharmacy, Xinjiang Key Laboratory of Clinical Drug Research, The First Affiliated Hospital of Xinjiang Medical University, Urumqi, 830013 China

**Keywords:** Anthocyanins, Atherosclerosis, Xinjiang wild cherry plum, *Prunus divaricata* Ledeb, Network pharmacology, Molecular docking

## Abstract

Atherosclerosis is a chronic vascular disease characterized by failure to resolve inflammation and forming plaque within the arterial wall. Atherosclerosis and its related cardiovascular diseases are the major causes of death worldwide. Our previous preliminary study showed that anthocyanin-rich extract (ACNE) from Xinjiang wild cherry plum (*Prunus divaricata* Ledeb) fruit peels exhibited anti-atherosclerotic effect. However, the potential mechanism of this health-beneficial effect remains unclear. Here, network pharmacology combined with molecular docking was used to tentatively address this issue. The ACNE mainly contains cyanidin, cyanidin 3-glucoside (Cy3Glu), Cyanidin 3-(6’’-acetylglucoside) (Cy3AcGlu), cyanidin 3-galactoside (Cy3Gal), cyanidin 3-xyloside (Cy3Xyl), and cyanidin 3-rutinoside (Cy3Rut). Seven key targets, EGFR, VEGFA, HSP90AA1, SRC, HIF1A, CXCR4 and IGF1R were identified from core protein-protein interaction (PPI) network. Anthocyanins interacting on key targets were initially demonstrated by molecular docking, particularly Cy3Rut and Cy3Xyl having highest affinity with most key targets. Biological function analysis suggested that key targets were involved in several biological processes, including positive regulation of cell migration, positive regulation of phosphorylation, inflammatory response, response to hypoxia, etc. The significantly enriched pathways, such as HIF-1 signaling pathway, calcium signaling pathway, macrophage stimulating protein MSP signaling network map, were closely related to atherosclerosis. Altogether, based on the comprehensive analysis and discussion, we revealed that TLR4/EGFR and IGF1R-CXCL12/CXCR4 pathways were at least partially implicated in the anti-atherosclerotic effects of anthocyanins through affecting inflammation, endothelial homeostasis, and foam cell formation. This study served as a theoretical basis for further validating the underlying anti-atherosclerotic mechanism of anthocyanins via in vitro and in vivo experiments.

**Supplementary Information:**

The online version contains supplementary material available at 10.1186/s40643-025-00900-w.

## Graphical Abstract

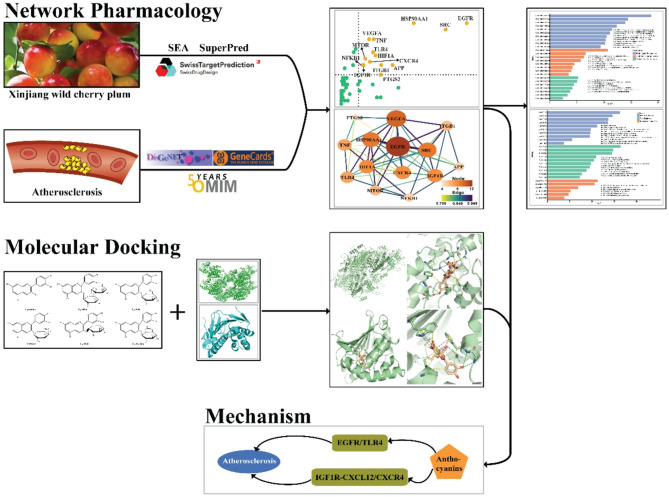

## Introduction

Atherosclerosis is a progressive disorder affecting the arterial wall of medium-to-large arteries. It is the underlying cause of cardiovascular diseases such as heart attack and stroke that are associated with high mortality and morbidity worldwide. It is reported that atherosclerotic cardiovascular disease (ASCVD) affects more than 500 million individuals globally and accounts for 19 million deaths annually (Li et al. 2023). Atherosclerosis is characterized by chronic unresolved inflammation and gradual arterial plaque deposition, with endothelial dysfunction as an initiating factor (Björkegren and Lusis 2022). Endothelial cells activated by oxidized lipids and proinflammatory stimuli express chemotactic factors and adhesion molecules, which attract monocytes to migrate into the subendothelial space, following differentiating into macrophages. In the arterial intima, monocyte-derived macrophages engulf oxidized low-density lipoprotein (oxLDL), resulting in lipid-laden foam cells formation and the development of fatty streaks, eventually becoming atherosclerotic plaques (Björkegren and Lusis 2022). These plaques make arterial lumen narrow and are prone to rupture or erosion, which readily causes catastrophic thrombosis in heart, brain or extremities, as a result of tissue infarction (Wolf and Ley 2019). Besides endothelial cells and macrophages, vascular smooth muscle cells, neutrophils and T cells have also been implicated in the pathogenesis and progression of atherosclerosis (Stroope et al. 2024).

Traditionally, hypertension, smoking, diabetes mellitus, obesity and so on have been recognized as risk factors of atheroma. Now, non-traditional drivers of atherosclerosis including disturbed sleep, physical inactivity, the microbiome, air pollution and environmental stress have gained attention (Libby 2021). The traditional and emerging risk factors are bridged by inflammatory pathways and leukocytes, collectively altering behavior of arterial wall cells. Over the past three decades, prevention and treatment of ASCVD were mainly concentrated on regimens regulating LDL-cholesterol via statins and PCSK9 (proprotein convertase subtilisin/kexin type 9)-blocking antibodies, and therapies targeting other traditional risk factors (Bergheanu et al. 2017; Li et al. 2022a, b). The cardiovascular mortality has decreased substantially, yet the global burden of cardiovascular disease remains huge. There is evidence that residual inflammatory risk remains significant even when LDL cholesterol regulated to target level, as well as being a stronger predictor of fatal and non-fatal events compared to residual cholesterol risk (Ridker et al. 2023; Verma et al. 2023). Given the primordial, persistent and indispensable role of inflammatory components in the pathogenesis of atherosclerosis, inflammatory pathways have been suggested as promising clinical targets for treating ASCVD (Engelen et al. 2022; Kong et al. 2022).

In China, there are a group of indigenous wide cherry plums (*Prunus divaricata* Ledeb) naturally growing in the Ili River Valley of the Western Tianshan Mountains, located at Huocheng County, Ili Kazakh Autonomous Prefecture of Xinjiang Uygur Autonomous Region (Wang et al. 2006). They produce wild fruits which can serve as food source for human being and other wild animals. These wild fruits possess high nutritional value and potential health benefits because of containing many nutraceutical and bioactive components such as polyphenols, flavonoids, polysaccharides, alimentary fiber, ascorbic acid, and minerals (Smanalieva et al. 2019; Shen et al. 2021). Among them, a subgroup of flavonoids, well known as anthocyanins, are present in the peels with high abundance. Anthocyanins are naturally occurring water-soluble vacuolar pigments, which give most higher plants (especially fruits, vegetables, flowers, and grains) brilliant colors with range from red-orange to blue-violet (Chen et al. 2023). Besides as natural pigments, anthocyanins are secondary metabolites protecting plants from biotic and abiotic stresses(Zhang and Zhu 2023). In structural sense, they contain anthocyanidin skeletons (C6-C3-C6) and sugar moieties, occasionally with acyl modification (Yang et al. 2023). There are more than 700 different anthocyanins, which predominantly assemble with six anthocyanidins including pelargonidin, cyanidin, delphinidin, peonidin, petunidin, malvidin and several common sugar residues such as glucose, ribose, xylose, galactose, rutinose (Yang et al. 2023). Diversity in their structure derives from the number and the position of the sugar moieties attached to aglycones (anthocyanidins) and the hydroxylation of aglycones and the acylation of sugar residues. Our previous study showed that the total anthocyanin content indicated as cyanidin 3-glucoside was 48% in the anthocyanins extract (ACNE) from Xinjiang wild cherry plum fruit peels (Shen et al. 2021), which was evaluated by a pH-differential method. The composition of ACNE analyzed by LC-MS/MS, mainly consist of cyanidin, cyanidin 3-glucoside, Cyanidin 3-(6’’-acetylglucoside), cyanidin 3-galactoside, cyanidin 3-xyloside, and cyanidin 3-rutinoside (Shen et al. 2021).

Chemical structure determines bioactivity. The promoting health effects of anthocyanins have gained extensive attention. Growing evidence demonstrates anthocyanins derived from different plant source have preventive and therapeutic effects against various chronic diseases, such as obesity, diabetes, nonalcoholic fatty liver disease, cardiovascular diseases, neurodegenerative diseases (Li et al. 2020; Franco-San Sebastián et al. 2023; Zaa et al. 2023; Mohammadi et al. 2024). It has been reported that anthocyanins exert protective effects on atherosclerosis. On one side, anthocyanins can reduce risk factors of atheroma, such as diabetes(Kozłowska and Nitsch-Osuch 2024), hypertension (Xin et al. 2024). On the other side, anthocyanins can exhibit anti-atherosclerotic action through their abilities of anti-inflammation, anti-oxidative stress, lipid regulation, endothelial protection (Danielewski et al. 2023; Szekeres et al. 2023). A study conducted by our group has shown that ACNE can significantly enhance anti-oxidant, anti-inflammatory capacity, and lower plasma lipids compared to atherosclerotic mice model (Shen et al. 2022). Additionally, a series of potential targets regarding lipid metabolism for ACNE’s anti-atherosclerotic action have also been identified by LC-MS/MS based-metabolomics analysis (Shen et al. 2022). Yet, the underlying anti-atherosclerotic mechanism of ACNE remains unclear. Here, we employ network pharmacology and molecular docking technologies to explore the molecular details on anti-atherosclerosis of anthocyanins derived from Xinjiang wild cherry plum fruit peels.

## Materials and methods

### Collection of anthocyanins and related targets

According to our analysis of anthocyanins-rich extract from Xinjiang wild cherry plum fruit peels, the anthocyanins composition included cyanidin, cyanidin 3-glucoside, cyanidin 3-(6’’-acetylglucoside), cyanidin 3-galactoside, cyanidin 3-xyloside, and cyanidin 3-rutinoside. The chemical SMILES of anthocyanins were obtained from PubChem database. The SMILES was used to retrieve target gene from Similarity ensemble approach (SEA), SuperPred and SwissTragetPrediction databases. The target genes were limited to *Homo sapiens* and unified by mapping to the HUGO Gene Nomenclature Committee (HGNC) gene symbol. Then, they were merged and removed duplicate genes.

### Collection of atherosclerosis related targets

Atherosclerosis related targets were collected from three databases, including DisGeNET (http://www.disgenet.org), GeneCards (https://www.genecards.org), Online Mendelian Inheritance in Man (OMIM, https://www.omim/org). “Atherosclerosis” was used as a searching keyword, and the species parameter was set as “*Homo sapiens*”. Moreover, targets from GeneCards and DisGeNET were further filtered out by relevance score > 1.0, score_gda ≥ 0.01, respectively. To avoid naming format bias, the obtained target genes were converted into standardized protein gene names via mapping to HGNC gene symbol. Subsequently, all targets were merged, and the duplicated targets were removed.

### Construction and analysis of the PPIs network

For constructing the protein-protein interactions (PPIs) network, firstly, the common targets between anthocyanins and atherosclerosis were obtained by intersecting two sets via the online tool InteractiVenn (http://www.interactivenn.net/), which can also use to perform the overlapping targets visualization through Venn plot. Subsequently, the PPIs network data was retrieved from STRING database (https://stringdb.org/) (accessed on 3 May 2023) with active interaction sources of textmining, experiments and databases, minimum required interaction score of 0.70 (high confidence), and organism specified as “*Homo sapiens*”. Finally, the PPIs network was constructed and visualized by Cytoscape V3.8.2 software. Network topological analysis was conducted by the Cytoscape plugin Centiscape2.2. To identify the key hub targets, another plugin cytoHubba0.1 was applied to comprehensively evaluate node centrality, with selection of MCC method.

### Gene ontology and pathway enrichment analysis

The gene ontology (GO) and pathway enrichment analysis was performed by online tools Metascape (http://metascape.org/gp/index.html), with defined organism “*Homo sapiens*”. The GO analysis covered three modules, biological processes (BP), cellular components (CC), molecular functions (MF). The pathway analysis included enrichment in KEGG, Reactome and WikiPathway databases. The most significant top10 enriched GO terms and pathways were displayed by bar plot, which were conducted with package ggplot2 (V3.4.3) under R environment (V4.2.0).

### Molecular docking

To investigate the interaction between anthocyanins and hub targets, molecular docking was performed by using AutoDock Vina V1.2.3. In brief, the 3D structures of anthocyanins were downloaded from PubChem in sdf format, which then were converted into pdb format by PyMOL V 2.5.5 software and processed by AutoDock Tools (ADT) V4.2 software, following outputting as pdbqt format. The crystal structures of hub targets were acquired from RCSB Protein Data Bank (PDB) in pdb format, with concentration on high resolution, completeness and human origin. Then, PyMOL and ADT were used to process the protein structure, including removing water, metal ions and original ligand, calculating charge, adding hydrogen, measuring grid box parameters, following the generation of pdbqt file. Subsequently, the interaction between the prepared ligands and proteins was evaluated by AutoDock Vina under CMD environment in Microsoft OS. The ligand with best docking conformation and the prepared protein structure were converted into one pdbqt file with PyMOL, and then used to predict possible interactions through online tool Protein-Ligand Interaction Profiler (PLIP, https://plip-tool.biotec.tu-dresden.de/plip-web/plip/index). Finally, the docking results were displayed by PyMOL and Adobe Illustrator CS6 V16.0.0.

## Results

### Screening of potential targets

**Fig. 1:**
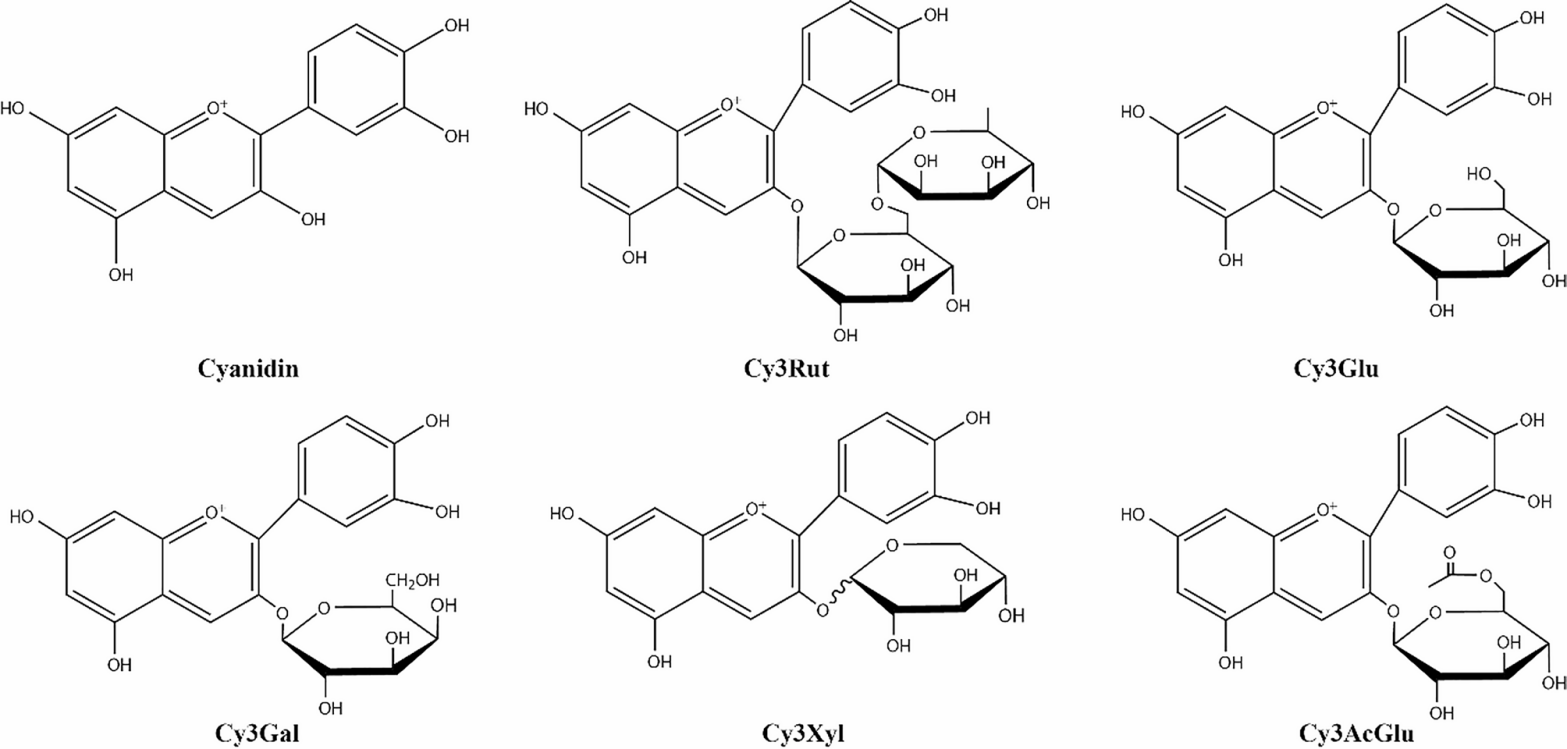
The chemical structure of anthocyanins presented in Xinjiang wild cherry plum peels. Cy3Rut: cyanidin 3-rutinoside, Cy3Glu: cyanidin 3-glucoside, Cy3Gal: cyanidin 3-galactoside, Cy3Xyl: cyanidin 3-xyloside, Cy3AcGlu: cyanidin 3-(6’’-acetylglucoside).

**Fig. 2:**
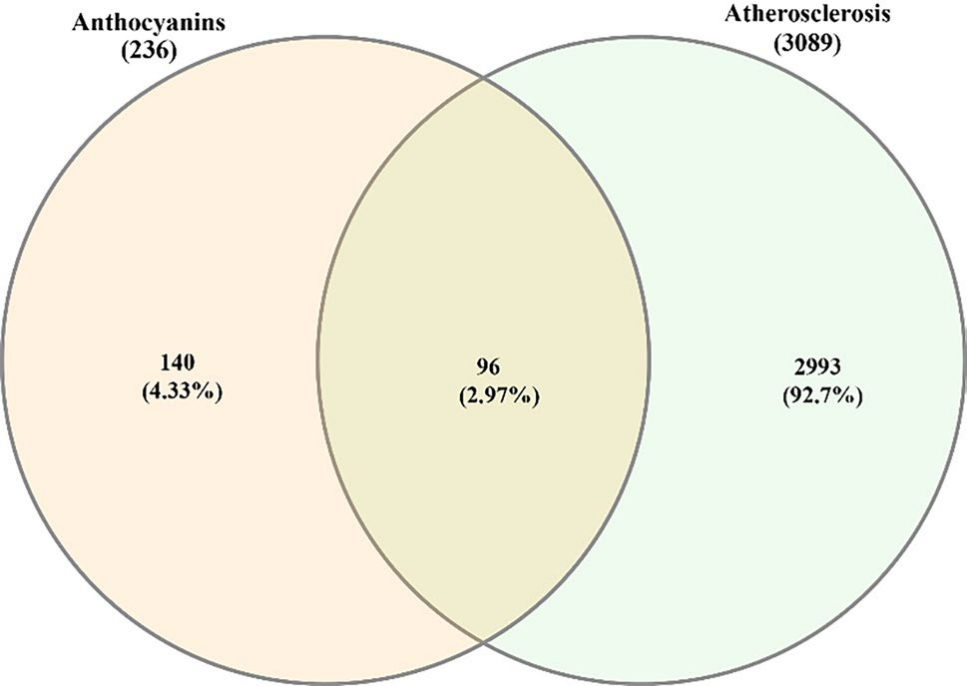
The Venn diagram of two target sets represented by anthocyanins and atherosclerosis, respectively. The two sets shared 96 targets. This Venn plot was performed by an online tool InteractiVenn.

A total of 236 dereplicated potential targets of six anthocyanins (Fig. 1) were retrieved from Similarity ensemble approach (SEA), SuperPred and SwissTragetPrediction databases. For disease targets, “atherosclerosis” was used to search against DisGeNET, GeneCards, OMIM, resulting in a total of 3943 pooled targets, and 3089 deduplicated targets. 96 common targets were obtained by intersecting two sets of targets related to anthocyanins and atherosclerosis, as illustrated in Fig. 2.

### Identification of hub targets

**Fig. 3:**
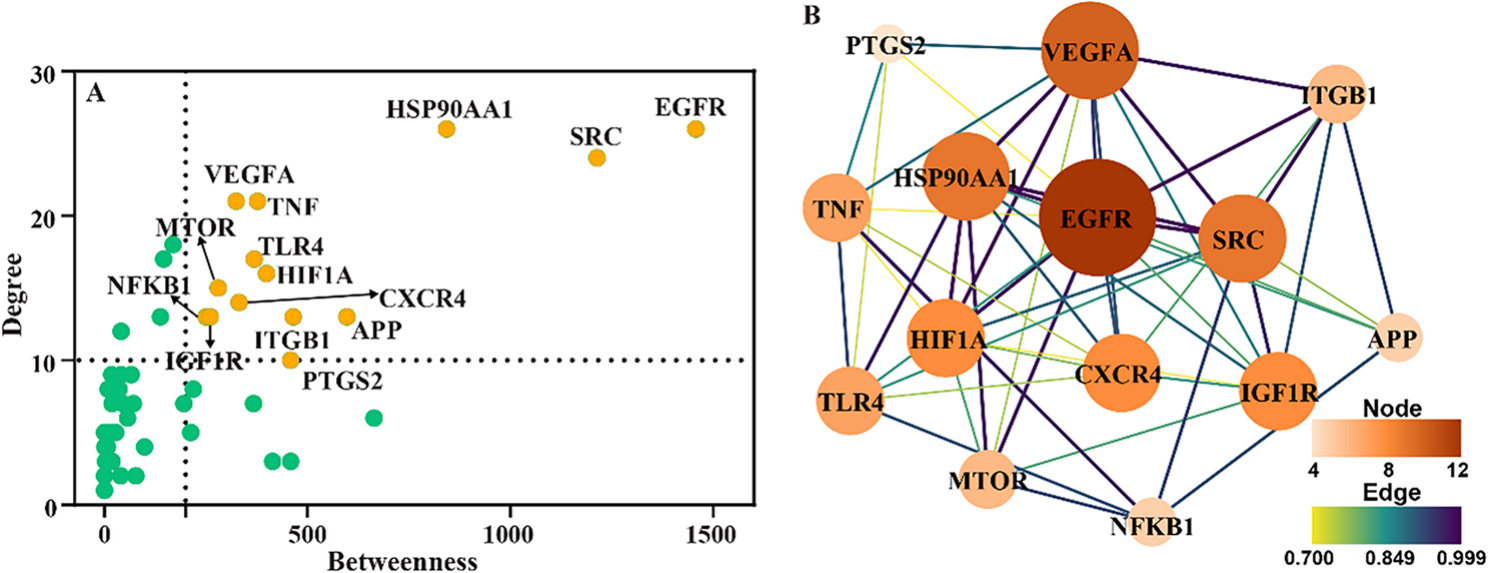
The PPI network and identification of hub targets. (**A**) Centrality of network nodes displayed by scatter plot. (**B**) Core PPI network screened from (**A**). The node size and color were represented with degree values. The edge size and color indicated combined score.

The PPIs data of 96 common targets was extracted from STRING and used to reconstructed the PPIs network by Cytoscape software. For identifying hub targets on the basis of betweenness and degree centrality, the network topological parameters were analyzed by the plugin Centiscape. Fourteen hub targets were screened by dgree ≥ 10 and betweenness ≥ 200 (Fig. 3A). The core PPIs network of 14 hub targets (Fig. 3B) were rebuilt and further analyzed by the plugin cytoHubba, with selection of MCC algorithm. The top 7 key hub targets with highest score including EGFR, VEGFA, IGF1R, HIF1A, HSP90AA1, CXCR4 and SRC were identified (Table S1).

### GO and pathway analysis

**Fig. 4:**
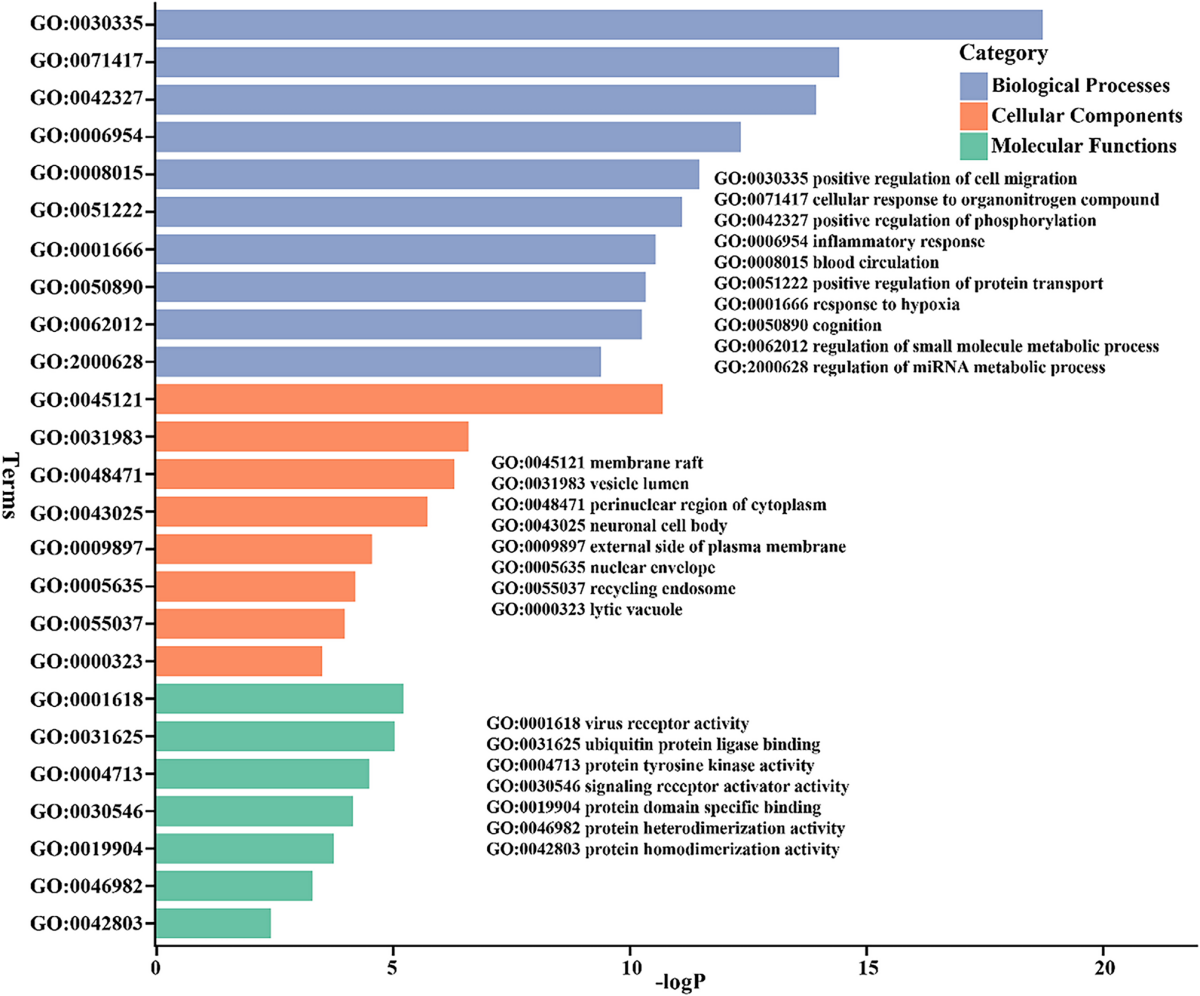
The GO enrichment analysis of 96 common targets by Metascape tool. The GO annotation included biological processes (BP), cellular components (CC), molecular functions (MF).

For understanding the molecular function of 96 common targets, GO enrichment analysis was conducted to identify the most enriched biological processes (BP), cellular components (CC) and molecular functions (MF). The top 10 GO terms were showed in Fig. 4. GO analysis showed that common targets mainly enriched in several biological processes, including positive regulation of cell migration (GO:0030335), cellular response to organonitrogen compound (GO:0071417), positive regulation of phosphorylation (GO:0042327), inflammatory response (GO:0006954), blood circulation (GO:0008015), positive regulation of protein transport (GO:0051222), response to hypoxia (GO:0001666). These biological processes might predominantly occur at the location of membrane raft (GO:0045121), vesicle lumen (GO:0031983), perinuclear region of cytoplasm (GO:0048471), neuronal cell body (GO:0043025), external side of plasma membrane (GO:0009897), nuclear envelope (GO:0005635), recycling endosome (GO:0055037). For MF enrichment, the 96 common targets mainly function in virus receptor activity (GO:0001618), ubiquitin protein ligase binding (GO:0031625), protein tyrosine kinase activity (GO:0004713), signaling receptor activator activity (GO:0030546), protein domain specific binding (GO:0019904).

**Fig. 5:**
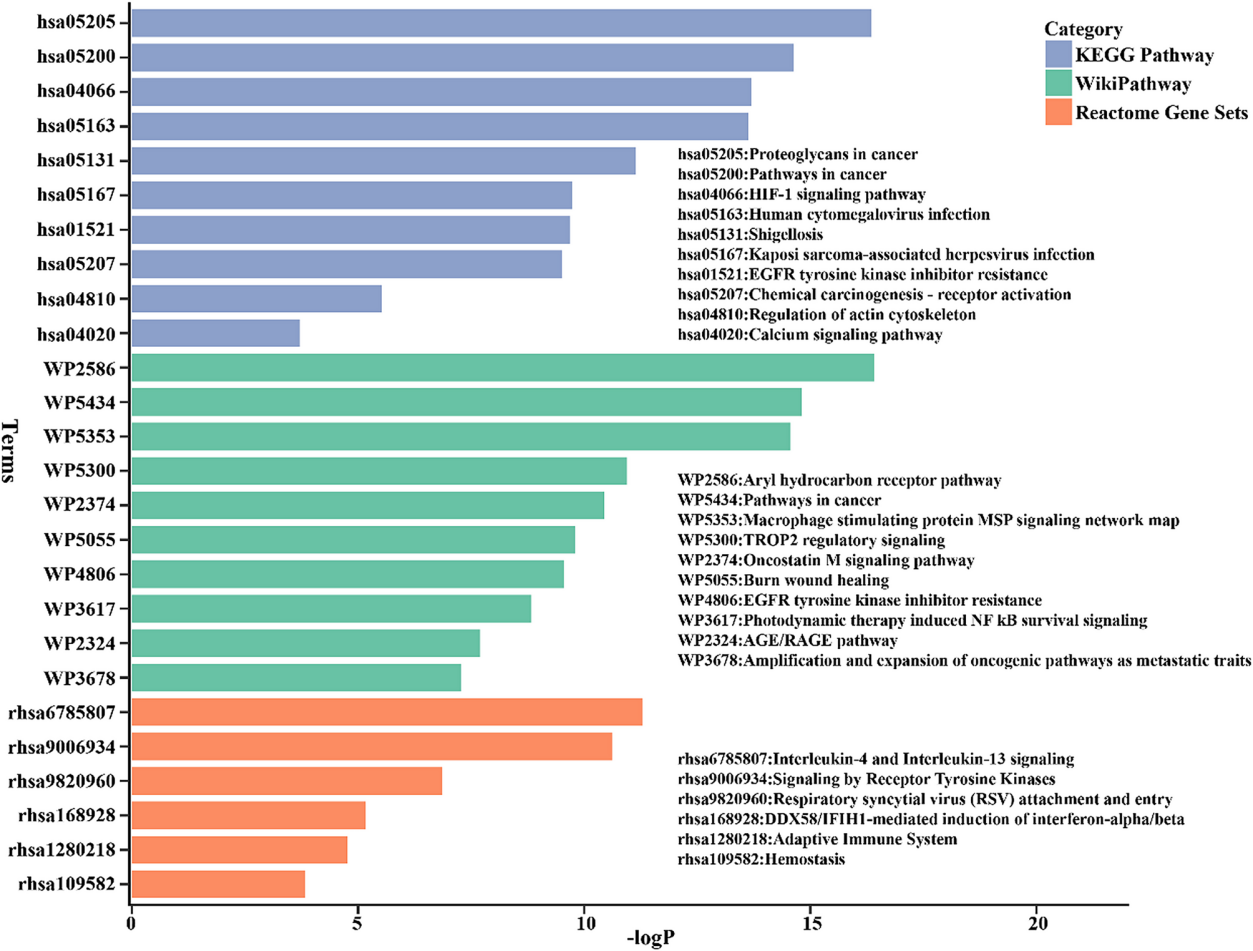
The pathway enrichment analysis of 96 common targets by Metascape tool. The pathway analysis covered three databases, including KEGG, Reactome, WikiPathways.

To explore the underlying molecular details on anti-atherosclerosis of anthocyanins, pathway enrichment analysis was performed by Metascape with covering of KEGG, Reactome and WikiPathway databases. As shown in Fig. 5, the common targets have implicated in atherosclerosis possibly through related pathways including HIF-1 signaling pathway (hsa04066), regulation of actin cytoskeleton (hsa04810), calcium signaling pathway (hsa04020), macrophage stimulating protein MSP signaling network map (WP5353), AGE/RAGE pathway (WP2324), interleukin-4 and interleukin-13 signaling (rhsa6785807), signaling by receptor tyrosine kinases (rhsa9006934), adaptive immune system (rhsa1280218), hemostasis (rhsa109582).

### Molecular docking analysis

In order to investigate the binding possibility between anthocyanins and key targets related to atherosclerosis, molecular docking was performed by using Autodock Vina tool. Considering docking scores varying with different docking parameters, three sets of grid box parameters covering active sites and multiple (2–5) crystal structures were employed to molecular docking. All docking cores were listed in Table S2. The key targets identified by network pharmacology combined with pivotal targets determined by our previous metabolomics analysis were used to dock with anthocyanins. On one hand, from the view of ligands, the optimal target of each anthocyanin was identified, as shown in Table 1. ENPP2 (PDB code: 5mhp, 8c3p) was the optimal docking protein of Cy3AcGlu and Cy3Xyl. EGFR (PDB code: 5gty) docked well with Cy3Rut and Cy3Gal, showing the highest affinity. HSP90AA1 (PDB code: 4bqg) and SRC (PDB code: 6ate) were the optimal targets of Cy3Glu and cyanidin, respectively, with the lowest docking score − 10.56, -9.70 kcal/mol. On the other hand, in the sight of targets, which anthocyanin docked best with each core target could be found out. The optimal docking results of 13 core target proteins (EGFR, VEGFA, HSP90AA1, SRC, HIF1A, CXCR4, IGF1R, AGPAT1, ENPP2, GPCPD1, LCAT and PPARG) and six anthocyanins were listed in Table 2. Cy3Rut and Cy3Xyl were the optimal docking ligands of most pivotal targets. Among them, Cy3Glu and HSP90AA1(PDB code: 4bqg), Cy3Rut and EGFR (PDB code: 5gty) docked well with each other, showing the optimal affinity (-10.56, -11.33 kcal/mol).

**Table 1:** The docking parameters and scores of anthocyanins with their optimal targets.

Ligand	Target	PDB code	Affinity (kcal/mol)	Grid_center (x, y, z)	Grid_size (x, y, z)
Cy3AcGlu	ENPP2	5mhp	-9.40	100.26, 1.49, 25.98	78, 88, 94
Cy3Glu	HSP90AA1	4bqg	-10.56	0.33, 14.86, 20.57	58, 60, 50
Cy3Xyl	ENPP2	8c3p	-9.56	29.38, 22.75, 100.87	94, 56,54
Cy3Rut	EGFR	5gty	-11.33	-27.44, 7.10, -71.52	78, 98, 88
Cy3Gal	EGFR	5gty	-10.03	-27.44, 7.10, -71.52	78, 98, 88
Cyanidin	SRC	6ate	-9.70	7.14, -35.77, 9.75	56, 40, 46

**Table 2:** The docking parameters and scores of hub targets with their optimal ligands.

Target	PDB code	Ligand	Affinity (kcal/mol)	Grid_center (x, y, z)	Grid_size (x, y, z)
EGFR	5gty	Cy3Rut	-11.33	-27.44, 7.10, -71.52	78, 98, 88
ENPP2	5mhp	Cy3Rut	-10.40	72.50, -3.70, 10.60	78, 88, 94
SRC	1byg	Cy3Rut	-10.30	28.92, 45.60, 15.16	102, 76, 108
PPARG	4ema	Cy3Rut	-10.00	12.81, -10.15, 32.41	78, 94, 96
CXCR4	3odu	Cy3Rut	-9.81	13.52, -12.06, 61.70	76, 92, 106
GPCPD1	2z0b	Cy3Rut	-9.34	42.34, 64.20, -12.11	68, 84, 94
AGPAT1	8erc	Cy3Rut	-9.20	101.57, 96.91, 99.27	98, 86, 110
LCAT	5txf	Cy3Rut	-9.08	83.71, 4.83, -0.94	96, 108, 88
PLA2G1B	6q42	Cy3Xyl	-9.41	7.60, 48.80, 131.70	86, 78, 96
IGF1R	1m7n	Cy3Xyl	-8.39	13.08, 17.52, 50.41	110, 126, 126
HIF1A	4h6j	Cy3Xyl	-6.65	14.10, -13.03, -34.94	56, 60, 40
HSP90AA1	4bqg	Cy3Glu	-10.56	0.33, 14.86, 20.57	58, 60, 50
VEGFA	1mkg	Cy3Gal	-7.90	-23.44, 43.65, -0.34	92, 112, 106

**Fig. 6:**
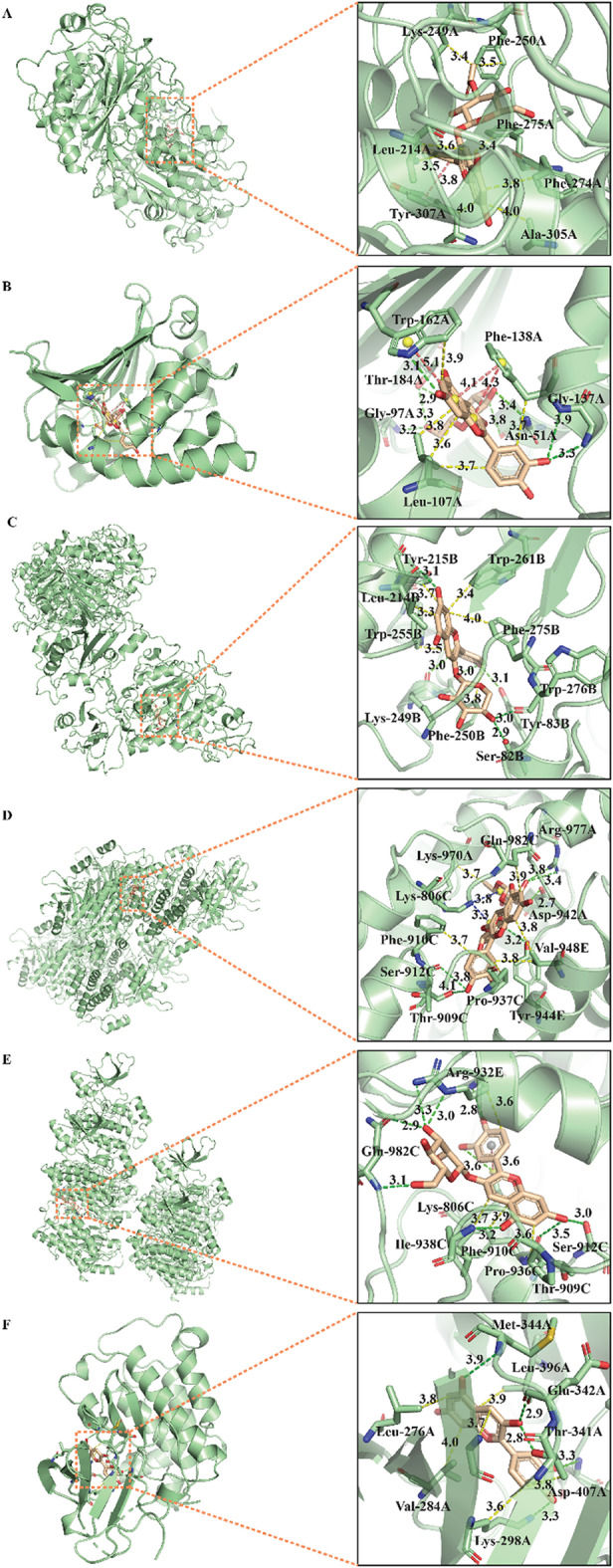
Molecular interaction and docking poses of anthocyanins with their optimal targets. (**A**) Cy3AcGlu-ENPP2(5mhp), (**B**) Cy3Glu-HSP90AA1(4bqg), (**C**) Cy3Xyl-ENPP2(8c3p), (**D**) Cy3Rut-EGFR(5gty), (**E**) Cy3Gal-EGFR(5gty) and (**F**) Cyanidin-SRC(6ate). Dotted lines with yellow, purplish red and green colors represented hydrophobic, hydrogen bond and π-stacking (π-cation) interactions, respectively.

**Fig. 7:**
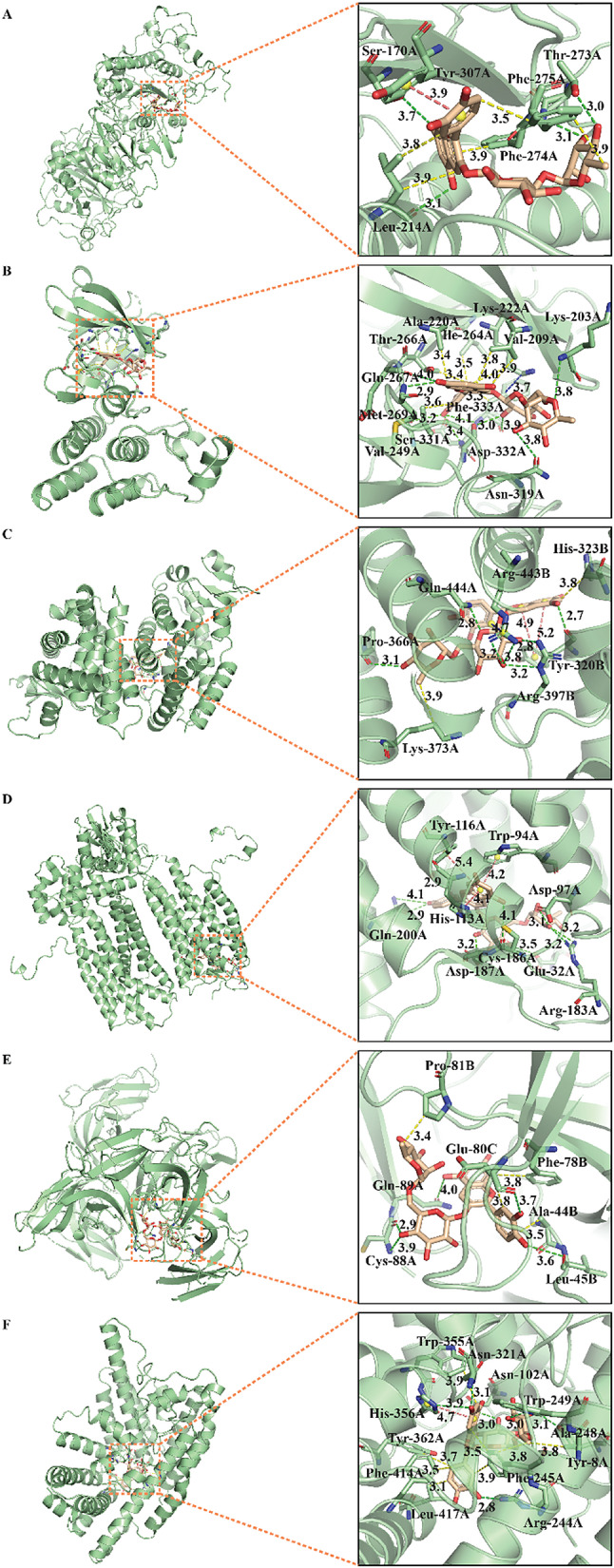
Molecular interaction and docking poses of key targets with their optimal ligands, Cy3Rut. (**A**) ENPP2(5mhp), (**B**) SRC(1byg), (**C**) PPARG(4ema), (**D**) CXCR4(3odu), (**E**) GPCPD1(2z0b) and (**F**) AGPAT1(8erc). Dotted lines with yellow, purplish red and green colors represented hydrophobic, hydrogen bond and π-stacking (π-cation) interactions, respectively.

For the purpose of further clarifying interacting details, the interacting type and sites of the complex comprising target and ligand with best docking conformation were predicted by online tool PLIP. The three-dimensional (3D) view of anthocyanin-target interaction was visualized and displayed in whole and local magnifying modes by PyMOL. The interactions between each anthocyanin and its optimal target were showed in Fig. 6. Likewise, the binding configuration of each key target and its optimal ligand was illustrated in Figs. 7 and S1. The predicted interactions primarily consisted of hydrophobic and hydrogen bonds interactions (see Figs. 6 and 7, S1, Tables S3, S4). The formation of above two main interactions was crucial for determining the specificity of candidates (ligands) combining with target proteins. Hydrogen bonds formed between the ligand and the amino acid residues within the active site facilitated the binding affinity and stability of the ligand-protein complex. Additionally, the binding specificity was further enhanced by the hydrophobic interactions between nonpolar regions of the ligand and the protein. Besides the above two main interactions, other critical interactions including salt bridges, π-stacking, π-cation were also observed in several anthocyanin-target complexes (Figs. 6 and 7, S1, Table S5, S6, S7). The salt bridges mainly occurred in side chain containing basic amino residues, such as LYS, ARG and HIS (Table S5).

## Discussions

Atherosclerosis is a lipid-driven chronic inflammatory vascular disease due to loss of vascular homeostasis, macrophage foam cell formation, and smooth muscle cell proliferation, resulting in progressive thickening of the blood vessel wall. It has been reported to be one of the main causes of death from cardiovascular disease. Its pathogenesis has implicated in multiple possible mechanisms, including lipid infiltrations, injury responses, inflammatory responses, immunological responses, and hemodynamic effects. Our previous study has demonstrated the anti-atherosclerosis activity of anthocyanins-rich extract from Xinjiang wild cherry plum fruit peels. Yet, the underlying mechanism is still elusive. In present study, we employ network pharmacology and molecular docking to clarify the relationship of anthocyanin-target-atherosclerosis.

The anthocyanins presented in Xinjiang wild cherry plum fruit peel mainly include cyanidin, cyanidin 3-glucoside (Cy3Glu), cyanidin 3-(6’’-acetylglucoside) (Cy3AcGlu), cyanidin 3-galactoside (Cy3Gal), cyanidin 3-xyloside (Cy3Xyl), and cyanidin 3-rutinoside (Cy3Rut). Among them, Cy3Glu and Cy3Rut derived from other plant sources have been suggested to exert antioxidant and anti-inflammatory effects on RAW264.7 cells in response to hydrogen peroxide and lipopolysaccharide (Jung et al. 2014). In addition, Cy3Glu can promote the efflux of cholesterol from the human aortic ECs (Wang et al. 2012a, b), regulate glucose metabolism and reduce hepatic and plasma triglyceride levels, adiposity (Jia et al. 2020). More recently, a study has shown that Cy3Glu can significantly alleviate the atherosclerotic lesion and inhibited platelet aggregation and activation in the setting of atherosclerotic inflammation (Yao et al. 2022). Cy3Glu activating liver X receptor α (LXRαWang et al. 2012a, b) and PPARs (with highest affinity for PPARα) (Jia et al. 2020) are responsible for regulation of cholesterol efflux and glucose metabolism, respectively.

In this study, EGFR, VEGFA, HSP90AA1, SRC, HIF1A, CXCR4 and IGF1R were identified as key hub targets, which linked anthocyanins with atherosclerosis. In line with other studies’ results (Li et al. 2022a, b; Sun et al. 2022; Liang et al. 2023), EGFR, HSP90AA1 and SRC were also recognized as key targets for treating atherosclerosis or acute myocardial infarction through network pharmacology and molecular docking. In these studies, in spite of HIF1A being not a key target, it was displayed in the core PPI network, which highlighted its important role in treatment of atherosclerosis or acute myocardial infarction. In our core PPI network (Fig. 3B), EGFR, epidermal growth factor receptor, has the greatest node degree and MCC score. Previous study was shown that Cy3Glu decreased EGFR phosphorylation (He et al. 2017). It has also been reported that disruption of toll-like receptor 4 (TLR4)/EGFR signaling pathway reduced inflammatory activity and foam cell formation, resulting in alleviating atherosclerosis (Wang et al. 2017). Coincidence with our results, TLR4 is also an important target exhibiting in the core PPI network (Fig. 3B). And there was experimental evidence supporting cyanidin decreasing the protein expression of TLR4 (Thummayot et al. 2018). Thus, based on the above analyses, it prompts that anthocyanins might exert anti-atherosclerotic effect via targeting on TLR4/EGFR pathway, at least partially. There is evidence that selective deleting or inhibiting EGFR in myeloid cells or CD4^+^ T cells limits atherosclerosis via reducing inflammatory factors production, such as TNF-α, IL-6, IL-4, IL-2, along with suppressing cytoskeletal rearrangements and also lipid uptake by macrophages and inducing anergy (Zeboudj et al. 2018a, b). The relationship of EGFR and inflammation is also evidenced by another study (Dubourg et al. 2023). The vascular endothelial growth factor (VEGF) family, as the crucial regulator of angiogenesis, lymphangiogenesis, lipid metabolism and inflammation, is involved in the development of atherosclerosis and further cardiovascular diseases (Dabravolski et al. 2022). It has been proved that macrophages with specifically expressed Vegfa accumulated in atherosclerotic lesions (Song et al. 2024). In vitro experimental results indicate that glycoside combinations of Buyang Huanwu decoction ameliorated atherosclerosis by inhibiting STAT3, HIF-1, and VEGF (Yan et al. 2023).

Heat shock protein 90 (HSP90) is a molecular chaperone, which is extensively involved in the regulation of protein folding and intracellular protein stability(Qi et al. 2022). It has been observed over-expression of HSP 90 family protein in atherosclerotic plaque, implying its close association with atherosclerosis. HSP90 participates in vascular inflammation and is related to diabetes-associated atherosclerosis (Ding et al. 2022). HSP90 inhibitors treatment can downregulate HSP90AA1 expression and ameliorate diabetes-driven atherosclerosis. It was evidenced by delaying plaque formation via inhibiting the migration and proliferation of vascular smooth muscle cells (Lazaro et al. 2017). This process is involved in activation of Nrf2 and suppression of NF-κB expression in plaques. Recently, a study reported that Hsp90 Cys521 mutation promoted nitric oxide bioavailability and alleviated endothelial adhesion, inflammation and oxidative stress in oxLDL-treated ECs through enhancing endothelial nitric oxide synthase activity and inhibiting NF-κB signaling (Zhao et al. 2022). Proto-oncogene tyrosine-protein kinase Src is a member of Src-family protein tyrosine kinases, which are promising targets for treating cardiovascular diseases (Zhai et al. 2021). Src has implicated in HDL acting as inducer of eNOS in endothelial cells (Abe et al. 2022). Src is recruited by SR-B1, and triggers autophosphorylation, leading to the phosphorylation and activation of AMPK (Abe et al. 2022). In addition, c-SRC facilitates the conversion of acetate to acetyl-CoA in cytosol via phosphorylating acetyl-CoA synthetase ACSS2 at Tyr530 and Tyr562 (Zhao et al. 2024). Hypoxia-inducible factor (HIF)-1α is expressed in various cell types of atherosclerotic lesions and is associated with lesional inflammation. Endothelial HIF-1α promotes atherosclerosis by triggering miR-19a mediated CXCL1 expression and monocyte adhesion (Akhtar et al. 2015). Inhibiting HIF-1α in adipocyte improves atherosclerosis through impairing ceramide generation (Wang et al. 2022). It is well known that the chemokine receptor CXCR4 (C-X-C chemokine receptor type 4) and its cognate ligand CXCL12 control cell homeostasis and trafficking. Previous study clearly established that vascular CXCR4 limited atherosclerosis by maintaining arterial integrity, preserving endothelial barrier function, and a normal contractile SMC phenotype (Döring et al. 2017). Similarly, another study demonstrated the protective effect of B-cell-specific CXCR4 on atherosclerosis (Döring et al. 2020). Moreover, it has been proved that Cy3Glu significantly downregulated CXCR4, CXCR7, and CCR5 in platelets and peripheral blood mononuclear cells and reduced plasma inflammatory chemokines CXCL4, CXCL5, CXCL7, CXCL12, CCL5 in atherosclerotic inflammation of ApoE^–/–^ mice (Yao et al. 2022).

Insulin-like growth factor-1 (IGF-1), a growth factor produced by a variety of cells and tissues throughout the body, is involved in tissue growth and differentiation, cell proliferation, survival, and anabolism via local (autocrine/paracrine) or systemic (endocrine) effects. It has been reported that macrophage specific IGF-1 overexpression reduced plaque macrophages, foam cells, and atherosclerotic burden. In macrophage-specific IGF-1 overexpressed mice, reduction in monocyte infiltration into plaque, decreased expression of CXCL12 (CXC chemokine ligand 12), and upregulation of ABCA1 (ATP-binding cassette transporter 1) were also observed. Furthermore, endothelial deficiency of IGF-1 receptor (IGF1R) caused endothelial barrier dysfunction and promoted atherosclerosis in ApoE-deficient mice. These results suggest that anthocyanins might exert protective effects on atherosclerosis through regulating IGF1R-CXCL12/CXCR4 pathway. Additionally, the six key targets, such as ENPP2, PPARG, GPCPD1, AGPAT1, LCAT, PLA2G1B, identified by metabolomics analysis of plasma samples from ApoE deficient mice treating with ACNE or corresponding control, are mainly linked to lipid metabolism (Shen et al. 2022).

Biological function analysis of common targets between anthocyanins and atherosclerosis revealed the significant enriched GO terms and pathways (Figs. 4 and 5). The biological processes and molecular functions, including positive regulation of cell migration (BP, GO:0030335), positive regulation of phosphorylation (BP, GO:0042327), inflammatory response (BP, GO:0006954), blood circulation (BP, GO:0008015), positive regulation of protein transport (BP, GO:0051222), response to hypoxia (BP, GO:0001666), protein tyrosine kinase activity (MF, GO:0004713), signaling receptor activator activity (MF, GO:0030546), protein domain specific binding (MF, GO:0019904), were closely related to key hub targets. For instance, autophosphorylation of SRC brings about the phosphorylation and activation of AMPK, which reflects positive regulation of phosphorylation (BP, GO:0042327). As described before, obviously, HIF1A, a key regulator in HIF-1 signaling pathway (KEGG, has04066), drives the progression of atherosclerosis. Besides HIF1A, EGFR, IGF1R, TLR4, VEGFA and HSP90AA1 are also enriched in HIF-1 signaling pathway. The pathway, regulation of actin cytoskeleton (KEGG, hsa04810), enriches three key hub targets, including EGFR, SRC and CXCR4. A study on flow-induced atherosclerotic mice model demonstrated that disturbed-flow induced a dramatic transition of ECs from atheroprotective phenotypes to pro-inflammatory cells, mesenchymal cells, hematopoietic stem cells phenotypes (Andueza et al. 2020). While Src family kinases play critical role in epithelial homeostasis (morphology and epithelial integrity) through regulation of actin cytoskeletal dynamics and cellular adhesions, which involves EMT (Ortiz et al. 2021). Recently, another study established that disturbed flow-induced atherosclerosis was dependent on integrin-actin cytoskeleton-NF-κB pathway (Fang et al. 2023). Thereby, regulation of actin cytoskeleton is closely related to atherosclerosis. The calcium signaling pathway (hsa04020) also involves three key hub targets EGFR, VEGFA, CXCR4. Calcium signaling plays pivotal function in atherosclerosis. This signaling can be triggered in endothelial cells exposed to external stimuli, such as fluid shear stress and ligand binding (Plank et al. 2006). Moreover, calcium deposition in the atherosclerotic artery lumen accelerates and solidifies plaque formation making the vessel narrow (Kalampogias et al. 2016). It has been suggested that VEGFA releases Ca^2+^ from IP3-sensitive stores and induces store-operated calcium influx via increasing the level of intracellular inositol 1,4,5-trisphosphate (IP3) (Faehling et al. 2002). VEGFA induced proliferation can be inhibited by decreasing extracellular or intracellular free Ca^2+^. CXCR4 can induce Ca^2+^ release through activating Gαq subunit, and further regulating protein kinase C (PKC) signaling (Beletkaia et al. 2016). EGFR is a receptor tyrosine kinase which can hydrolyze phosphatidylinositol 4,5-bisphosphate (PI(4,5)P2) within the nucleus, causing Ca^2+^ release from the nucleoplasmic reticulum by IP3 receptors (de Miranda et al. 2019). Other significantly enriched pathways are not further discussed because of limited paper length.

Molecular docking results uncover the possible binding pattern of anthocyanins and targets, each target’s best ligand (anthocyanin, shown in Table 2). Most targets dock optimally with Cy3Rut, next with Cy3Xyl, as expressed by docking score (affinity, kcal/mol). The results suggest that binding occurs in one anthocyanin mapping to multiple targets, or multiple anthocyanins mapping to one target via common hydrophobic and hydrogen bonds interactions, occasionally salt bridges, π-stacking, π-cation.

So far, studies about Xinjiang wild cherry plum are mainly focused on the plant field, such as genetic diversity (Zhao et al. 2015), evaluation of phytochemicals and antioxidant activities (Wang et al. 2012a, b; Liu et al. 2019; Shen et al. 2021). The researches on pharmacological effect of anthocyanins from Xinjiang wild cherry plum are scarce. However, there are extensive studies on anthocyanins from other natural sources, which have shown efficacy against multiple diseases including cancer (breast cancer (Nava-Ochoa et al. 2025), non-small cell lung cancer (Papavassiliou et al. 2024), metabolic syndrome (Liu et al. 2025), nonalcoholic fatty liver disease (Dias et al. 2025), Alzheimer’s disease(Bayazid and Lim 2024), hypertensive and atherosclerosis (Xin et al. 2024), underlying the primary antioxidant, anti-inflammatory activities, regulation of lipid disorder and gut microbiota dysbiosis. For example, anthocyanins from dark sweet cherry exhibit potential to inhibit drug resistance in 4T1 breast cancer cells (Nava-Ochoa et al. 2025). Leptin pathway is clarified as a crucial target for anthocyanins to protect against metabolic syndrome (Liu et al. 2025). A previous investigation indicated that the purified anthocyanin from *Lycium ruthenicum* showcased ameliorative effect on atherosclerosis in rats through synergistic modulation of the gut microbiota and NF-κB/SREBP-2 pathways (Luo et al. 2019). Based on the multiple actives and human beneficial effects, anthocyanins can be used as biological pesticides or feed additives in agricultural production, natural pigments, food packaging or functional foods in food industry, adjuvant therapy of disease in medical field.

### Limitations

Although the strategy of combining network pharmacology with molecular docking is highly of potential in prediction of bioactive components and elucidating action of mode, there are still some limitations. The accuracy of prediction and analyzing results is profoundly influenced by quality of biological data (including time validity, accuracy, completeness, availability of database, etc.), computational complexities (including algorithms and precision of scoring function, etc.) and biological system redundancy. In addition, the conduction of docking usually requires targets with known crystal structure and rigid model. The strategy also faces challenges from guidelines, regulatory associated with clinical trials and drug approval. Here, in spite of the strategy reveals the anti-atherosclerotic mechanism of anthocyanins from Xinjiang wild cherry plum fruit peels, there is still lack of validatory experimental evidence. This is also a main defect in this study. Next, we will consider establishment of their interactions via experiments or assays.

## Conclusion

In this study, network pharmacology and molecular docking technologies are used to unravel the mechanism underlying anti-atherosclerotic effect of anthocyanins from Xinjiang wild cherry plum fruit peels. Our results have suggested that Cy3Rut, Cy3Xyl, Cy3Glu, Cy3Gal might interact on EGFR, VEGFA, HSP90AA1, SRC, HIF1A, CXCR4, IGF1R and lipid metabolism related targets for exerting anti-atherosclerotic effect. The interactions between anthocyanins and key hub targets are initially demonstrated by molecular docking analysis. The functions of these key hub targets can be preliminary interpreted by significantly enriched GO and pathway annotations. In sum, based on the comprehensive discussion, we propose that anthocyanins, particularly Cy3Rut and Cy3Xyl, play anti-atherosclerotic function by affecting inflammation, endothelial homeostasis, and foam cell formation via regulating TLR4/EGFR pathway, IGF1R-CXCL12/CXCR4 pathway, at least partially. This study deepens our understanding in anti-atherosclerotic effect of anthocyanins and offers scientific basis for developing effective therapeutic strategies of atherosclerosis. Also, this will facilitate the exploitation and utilization of wild bioresource—Xinjiang wild cherry plum. Owing to the limitations of network pharmacology and molecular docking, next in vitro and in vivo experiments will be conducted to investigate the effects of Cy3Rut and Cy3Xyl on TLR4/EGFR, IGF1R-CXCL12/CXCR4 pathways in atherosclerotic cell and animal models.

## Electronic supplementary material

Below is the link to the electronic supplementary material.

Supplementary Material 1

## Data Availability

The datasets utilized and examined in the present study can be obtained upon a reasonable request from the corresponding author.

## Abbreviations

ACNE: Anthocyanin-rich extract
Cy3Glu: Cyanidin 3-glucoside
Cy3AcGlu: Cyanidin 3-(6’’-acetylglucoside)
Cy3Gal: Cyanidin 3-galactoside
Cy3Xyl: Cyanidin 3-xyloside
Cy3Rut: Cyanidin 3-rutinoside
EGFR: Epidermal growth factor receptor
VEGFA: Vascular endothelial growth factor A
HSP90AA1: Heat shock protein 90 alpha family class A member 1
SRC: Proto-oncogene, non-receptor tyrosine kinase
HIF1A: Hypoxia-inducible factor 1-alpha
CXCR4: C-X-C chemokine receptor type 4
IGF1R: Insulin-like growth factor 1 receptor
ASCVD: Atherosclerotic cardiovascular disease
oxLDL: Oxidized low-density lipoprotein
PCSK9: Proprotein convertase subtilisin/kexin type 9
TLR4: Toll-like receptor 4
MMP9: Matrix metalloproteinase-9
GO: Gene ontology
KEEG: Kyoto encyclopedia of genes and genomes

## References

[CR1] Abe RJ, Abe J-i, Nguyen MTH, Olmsted-Davis EA, Mamun A, Banerjee P, Cooke JP, Fang L, Pownall H, Le N-T (2022) Free cholesterol bioavailability and atherosclerosis. Curr Atheroscler Rep 24:323–336. 10.1007/s11883-022-01011-z 35332444 10.1007/s11883-022-01011-z PMC9050774

[CR2] Akhtar S, Hartmann P, Karshovska E, Rinderknecht FA, Subramanian P, Gremse F, Grommes J, Jacobs M, Kiessling F, Weber C, Steffens S, Schober A (2015) Endothelial hypoxia-inducible factor-1α promotes atherosclerosis and monocyte recruitment by upregulating microRNA-19a. Hypertension 66:1220–1226. 10.1161/hypertensionaha.115.05886 26483345 10.1161/HYPERTENSIONAHA.115.05886

[CR3] Andueza A, Kumar S, Kim J, Kang D-W, Mumme HL, Perez JI, Villa-Roel N, Jo H (2020) Endothelial reprogramming by disturbed flow revealed by single-cell RNA and chromatin accessibility study. Cell Rep 33:108491. 10.1016/j.celrep.2020.108491 33326796 10.1016/j.celrep.2020.108491 PMC7801938

[CR4] Bayazid AB, Lim BO (2024) Therapeutic effects of plant anthocyanin against Alzheimer’s disease and modulate gut health, short-chain fatty acids. Nutrients 16:1554. 10.3390/nu16111554 10.3390/nu16111554 PMC11173718 38892488

[CR5] Beletkaia E, Fenz SF, Pomp W, Snaar-Jagalska BE, Hogendoorn PWC, Schmidt T (2016) CXCR4 signaling is controlled by immobilization at the plasma membrane. Biochimica et biophysica acta (BBA) - Mol Cell Res 1863:607–616. 10.1016/j.bbamcr.2015.12.020 10.1016/j.bbamcr.2015.12.020 26748383

[CR6] Bergheanu SC, Bodde MC, Jukema JW (2017) Pathophysiology and treatment of atherosclerosis: current view and future perspective on lipoprotein modification treatment. Neth Heart J 25:231–242. 10.1007/s12471-017-0959-2 28194698 10.1007/s12471-017-0959-2 PMC5355390

[CR7] Björkegren JLM, Lusis AJ (2022) Atherosclerosis: recent developments. Cell 185:1630–1645. 10.1016/j.cell.2022.04.004 35504280 10.1016/j.cell.2022.04.004 PMC9119695

[CR8] Chen K, Kortesniemi MK, Linderborg KM, Yang B (2023) Anthocyanins as promising molecules affecting energy homeostasis, inflammation, and gut microbiota in type 2 diabetes with special reference to impact of acylation. J Agric Food Chem 71:1002–1017. 10.1021/acs.jafc.2c05879 36515085 10.1021/acs.jafc.2c05879 PMC9853865

[CR9] Dabravolski SA, Khotina VA, Omelchenko AV, Kalmykov VA, Orekhov AN (2022) The role of the VEGF family in atherosclerosis development and its potential as treatment targets. Int J Mol Sci 23:931. 10.3390/ijms23020931 10.3390/ijms23020931 PMC8781560 35055117

[CR10] Danielewski M, Gomułkiewicz A, Kucharska AZ, Matuszewska A, Nowak B, Piórecki N, Trocha M, Szandruk-Bender M, Jawień P, Szeląg A, Dzięgiel P, Sozański T (2023) Cornelian Cherry (*Cornus Mas* L.) iridoid and anthocyanin-rich extract reduces various oxidation, inflammation, and adhesion markers in a cholesterol-rich diet rabbit model. Int J Mol Sci 24:3890. 10.3390/ijms24043890 10.3390/ijms24043890 PMC9959706 36835296

[CR11] de Miranda MC, Rodrigues MA, de Angelis Campos AC, Faria J, Kunrath-Lima M, Mignery GA, Schechtman D, Goes AM, Nathanson MH, Gomes DA (2019) Epidermal growth factor (EGF) triggers nuclear calcium signaling through the intranuclear phospholipase Cδ-4 (PLCδ4). J Biol Chem 294:16650–16662. 10.1074/jbc.RA118.006961 31537645 10.1074/jbc.RA118.006961 PMC6851314

[CR12] Dias KA, Oliveira LA, Pereira SMS, Abrantes LCS, Vicente L, Gonçalves RV, Della Lucia CM (2025) Anti-inflammatory and antioxidant effects of anthocyanins in nonalcoholic fatty liver disease (NAFLD): a systematic review of in vivo studies. Crit Rev Food Sci Nutr 1–18. 10.1080/10408398.2025.2472882 10.1080/10408398.2025.2472882 40045715

[CR13] Ding X, Meng C, Dong H, Zhang S, Zhou H, Tan W, Huang L, He A, Li J, Huang J, Li W, Zou F, Zou M (2022) Extracellular Hsp90α, which participates in vascular inflammation, is a novel serum predictor of atherosclerosis in type 2 diabetes. BMJ Open Diabetes Res Care 10:e002579. 10.1136/bmjdrc-2021-002579 10.1136/bmjdrc-2021-002579 PMC8804642 35091448

[CR15] Döring Y, Noels H, van der Vorst EPC, Neideck C, Egea V, Drechsler M, Mandl M, Pawig L, Jansen Y, Schröder K, Bidzhekov K, Megens RTA, Theelen W, Klinkhammer BM, Boor P, Schurgers L, van Gorp R, Ries C, Kusters PJH, van der Wal A, Hackeng TM, Gäbel G, Brandes RP, Soehnlein O, Lutgens E, Vestweber D, Teupser D, Holdt LM, Rader DJ, Saleheen D, Weber C (2017) Vascular CXCR4 limits atherosclerosis by maintaining arterial integrity: evidence from mouse and human studies. Circulation 136:388–403. 10.1161/circulationaha.117.027646 28450349 10.1161/CIRCULATIONAHA.117.027646 PMC5777319

[CR14] Döring Y, Jansen Y, Cimen I, Aslani M, Gencer S, Peters LJF, Duchene J, Weber C, van der Vorst EPC (2020) B-cell-specific CXCR4 protects against atherosclerosis development and increases plasma IgM levels. Circul Res 126:787–788. 10.1161/circresaha.119.316142 10.1161/CIRCRESAHA.119.316142 32078474

[CR16] Dubourg V, Schwerdt G, Schreier B, Kopf M, Mildenberger S, Benndorf RA, Gekle M (2023) EGFR activation differentially affects the inflammatory profiles of female human aortic and coronary artery endothelial cells. Sci Rep 13:22827. 10.1038/s41598-023-50148-7 38129563 10.1038/s41598-023-50148-7 PMC10739936

[CR17] Engelen SE, Robinson AJB, Zurke YX, Monaco C (2022) Therapeutic strategies targeting inflammation and immunity in atherosclerosis: how to proceed? Nat Rev Cardiol 19:522–542. 10.1038/s41569-021-00668-4 35102320 10.1038/s41569-021-00668-4 PMC8802279

[CR18] Faehling M, Kroll J, Föhr KJ, Fellbrich G, Mayr U, Trischler G, Waltenberger J (2002) Essential role of calcium in vascular endothelial growth factor A-induced signaling: mechanism of the antiangiogenic effect of carboxyamidotriazole. FASEB J 16:1805–1807. 10.1096/fj.01-0938fje 12354692 10.1096/fj.01-0938fje

[CR19] Fang F, Feng T, Li J, Zhang H, Wang Q, Chen Y, Wang G, Shen Y, Liu X (2023) Cathepsin K contributed to disturbed flow-induced atherosclerosis is dependent on integrin-actin cytoskeleton-NF-κB pathway. Genes Dis 10:583–595. 10.1016/j.gendis.2022.03.020 37223522 10.1016/j.gendis.2022.03.020 PMC10201601

[CR20] Franco-San Sebastián D, Alaniz-Monreal S, Rabadán-Chávez G, Vázquez-Manjarrez N, Hernández-Ortega M, Gutiérrez-Salmeán G (2023) Anthocyanins: potential therapeutic approaches towards obesity and diabetes mellitus type 2. Molecules 28:1237. 10.3390/molecules28031237 10.3390/molecules28031237 PMC9919338 36770906

[CR21] He Y, Hu Y, Jiang X, Chen T, Ma Y, Wu S, Sun J, Jiao R, Li X, Deng L, Bai W (2017) Cyanidin-3-O-glucoside inhibits the UVB-induced ROS/COX-2 pathway in HaCaT cells. J Photochem Photobiol B 177:24–31. 10.1016/j.jphotobiol.2017.10.006 29031211 10.1016/j.jphotobiol.2017.10.006

[CR22] Jia Y, Wu C, Kim Y-S, Yang SO, Kim Y, Kim J-S, Jeong M-Y, Lee JH, Kim B, Lee S, Oh H-S, Kim J, So M-Y, Yoon YE, Thach TT, Park TH, Lee S-J (2020) A dietary anthocyanin cyanidin-3-O-glucoside binds to PPARs to regulate glucose metabolism and insulin sensitivity in mice. Commun Biology 3:514. 10.1038/s42003-020-01231-6 10.1038/s42003-020-01231-6 PMC7501857 32948821

[CR23] Jung H, Kwak H-K, Hwang KT (2014) Antioxidant and antiinflammatory activities of cyanidin-3-glucoside and cyanidin-3-rutinoside in hydrogen peroxide and lipopolysaccharide-treated RAW264.7 cells. Food Sci Biotechnol 23:2053–2062. 10.1007/s10068-014-0279-x

[CR24] Kalampogias A, Siasos G, Oikonomou E, Tsalamandris S, Mourouzis K, Tsigkou V, Vavuranakis M, Zografos T, Deftereos S, Stefanadis C, Tousoulis D (2016) Basic mechanisms in atherosclerosis: the role of calcium. Med Chem 12:103–113. 10.2174/1573406411666150928111446 26411606 10.2174/1573406411666150928111446

[CR25] Kong P, Cui ZY, Huang XF, Zhang DD, Guo RJ, Han M (2022) Inflammation and atherosclerosis: signaling pathways and therapeutic intervention. Signal Transduct Target Therapy 7:131. 10.1038/s41392-022-00955-7 10.1038/s41392-022-00955-7 PMC9033871 35459215

[CR26] Kozłowska A, Nitsch-Osuch A (2024) Anthocyanins and type 2 diabetes: an update of human study and clinical trial. Nutrients 16:1674. 10.3390/nu16111674 10.3390/nu16111674 PMC11174612 38892607

[CR27] Lazaro I, Oguiza A, Recio C, Lopez-Sanz L, Bernal S, Egido J, Gomez-Guerrero C (2017) Interplay between HSP90 and Nrf2 pathways in diabetes-associated atherosclerosis. Clin Investig Arterioscler 29:51–59. 10.1016/j.arteri.2016.10.003 28188022 10.1016/j.arteri.2016.10.003

[CR30] Li X, Shi Z, Zhu Y, Shen T, Wang H, Shui G, Loor JJ, Fang Z, Chen M, Wang X, Peng Z, Song Y, Wang Z, Du X, Liu G (2020) Cyanidin-3-O-glucoside improves non-alcoholic fatty liver disease by promoting PINK1-mediated mitophagy in mice. Br J Pharmacol 177:3591–3607. 10.1111/bph.15083 32343398 10.1111/bph.15083 PMC7348088

[CR28] Li H, Gao L, Shao H, Li B, Zhang C, Sheng H, Zhu L (2022a) Elucidation of active ingredients and mechanism of action of hawthorn in the prevention and treatment of atherosclerosis. J Food Biochem 46:e14457. 10.1111/jfbc.14457 36200679 10.1111/jfbc.14457

[CR29] Li L, Liu S, Tan J, Wei L, Wu D, Gao S, Weng Y, Chen J (2022b) Recent advance in treatment of atherosclerosis: key targets and plaque-positioned delivery strategies. J Tissue Eng 13:20417314221088509. 10.1177/20417314221088509 35356091 10.1177/20417314221088509 PMC8958685

[CR31] Li Y, Cao GY, Jing WZ, Liu J, Liu M (2023) Global trends and regional differences in incidence and mortality of cardiovascular disease, 1990–2019: findings from 2019 global burden of disease study. Eur J Prev Cardiol 30:276–286. 10.1093/eurjpc/zwac285 36458973 10.1093/eurjpc/zwac285

[CR32] Liang D, Yixuan D, Chang L, Jingjing S, Sihai Z, Jie D (2023) Mechanism of *Artemisia annua* L. in the treatment of acute myocardial infarction: network pharmacology, molecular docking and in vivo validation. Mol Diversity 28:3225-3242. 10.1007/s11030-023-10750-3 10.1007/s11030-023-10750-3 37898972

[CR33] Libby P (2021) The changing landscape of atherosclerosis. Nature 592:524–533. 10.1038/s41586-021-03392-8 33883728 10.1038/s41586-021-03392-8

[CR35] Liu X, Liu W, Ge Y, Liu T, An X (2019) Determination of polyphenol content and antioxidant activity in *Prunus Cerasifera* peel. J Yili Normal University(Natural Sci Edition) 13:48–53.

[CR34] Liu M, Li S, Guan M, Bai S, Bai W, Jiang X (2025) Leptin pathway is a crucial target for anthocyanins to protect against metabolic syndrome. Crit Rev Food Sci Nutr 65:2046–2061. 10.1080/10408398.2024.2323093 38567995 10.1080/10408398.2024.2323093

[CR36] Luo Y, Fang J-L, Yuan K, Jin S-H, Guo Y (2019) Ameliorative effect of purified anthocyanin from *Lycium ruthenicum* on atherosclerosis in rats through synergistic modulation of the gut microbiota and NF-κB/SREBP-2 pathways. J Funct Foods 59:223–233. 10.1016/j.jff.2019.05.038

[CR37] Mohammadi N, Farrell M, O’Sullivan L, Langan A, Franchin M, Azevedo L, Granato D (2024) Effectiveness of anthocyanin-containing foods and nutraceuticals in mitigating oxidative stress, inflammation, and cardiovascular health-related biomarkers: a systematic review of animal and human interventions. Food Funct 15:3274–3299. 10.1039/d3fo04579j 38482946 10.1039/d3fo04579j

[CR38] Nava-Ochoa A, Mertens-Talcott SU, Talcott ST, Noratto GD (2025) Dark sweet Cherry (*Prunus avium* L.) juice phenolics rich in anthocyanins exhibit potential to inhibit drug resistance mechanisms in 4T1 breast cancer cells via the drug metabolism pathway. Curr Issues Mol Biol 47:213. 10.3390/cimb47030213 10.3390/cimb47030213 PMC11941269 40136467

[CR39] Ortiz MA, Mikhailova T, Li X, Porter BA, Bah A, Kotula L (2021) Src family kinases, adaptor proteins and the actin cytoskeleton in epithelial-to-mesenchymal transition. Cell Communication Signaling: CCS 19:67. 10.1186/s12964-021-00750-x 34193161 10.1186/s12964-021-00750-x PMC8247114

[CR40] Papavassiliou KA, Sofianidi AA, Papavassiliou AG (2024) Anthocyanins in non-small cell lung cancer (NSCLC) treatment and prevention. Nutrients 16:1458. 10.3390/nu16101458 10.3390/nu16101458 PMC11124329 38794696

[CR41] Plank MJ, Wall DJ, David T (2006) Atherosclerosis and calcium signalling in endothelial cells. Prog Biophys Mol Biol 91:287–313. 10.1016/j.pbiomolbio.2005.07.005 16171849 10.1016/j.pbiomolbio.2005.07.005

[CR42] Qi S, Yi G, Yu K, Feng C, Deng S (2022) The role of HSP90 inhibitors in the treatment of cardiovascular diseases. Cells 11:3444. 10.3390/cells11213444 10.3390/cells11213444 PMC9657168 36359837

[CR43] Ridker PM, Bhatt DL, Pradhan AD, Glynn RJ, MacFadyen JG, Nissen SE (2023) Inflammation and cholesterol as predictors of cardiovascular events among patients receiving Statin therapy: a collaborative analysis of three randomised trials. Lancet 401:1293–1301. 10.1016/s0140-6736(23)00215-5 36893777 10.1016/S0140-6736(23)00215-5

[CR45] Shen J, Zhang P, Zhang X, Yao J, Chang JM (2021) Extraction process and composition identification of anthocyanins from Xinjiang wild *Prunus cerasifera* fruit peel. Pak J Pharm Sci 34:2409–2415. 35039252

[CR44] Shen J, Li X, Zhang X, Li Z, Abulaiti G, Liu Y, Yao J, Zhang P (2022) Effects of Xinjiang wild cherry plum (*Prunus divaricata* Ledeb) anthocyanin-rich extract on the plasma metabolome of atherosclerotic apoE-deficient mice fed a high-fat diet. Front Nutr 9:923699. 10.3389/fnut.2022.923699 35958261 10.3389/fnut.2022.923699 PMC9358619

[CR46] Smanalieva J, Iskakova J, Oskonbaeva Z, Wichern F, Darr D (2019) Determination of physicochemical parameters, phenolic content, and antioxidant capacity of wild cherry plum (*Prunus divaricata* Ledeb.) from the walnut-fruit forests of Kyrgyzstan. Eur Food Res Technol 245:2293–2301. 10.1007/s00217-019-03335-8

[CR47] Song YJ, Ma Y, Meng T, Zhuang T, Ruan CC, Li Y, Zhang GN (2024) The characteristics of macrophage heterogeneity in atherosclerotic aortas. J Cardiovasc Transl Res 17:153–166. 10.1007/s12265-023-10434-1 37713049 10.1007/s12265-023-10434-1

[CR48] Stroope C, Nettersheim FS, Coon B, Finney AC, Schwartz MA, Ley K, Rom O, Yurdagul A Jr (2024) Dysregulated cellular metabolism in atherosclerosis: mediators and therapeutic opportunities. Nat Metabolism 6:617–638. 10.1038/s42255-024-01015-w 10.1038/s42255-024-01015-w PMC11055680 38532071

[CR49] Sun T, Quan W, Peng S, Yang D, Liu J, He C, Chen Y, Hu B, Tuo Q (2022) Network pharmacology-based strategy combined with molecular docking and in vitro validation study to explore the underlying mechanism of Huo Luo Xiao Ling Dan in treating atherosclerosis. Drug Des Devel Ther 16:1621–1645. 10.2147/dddt.s357483 35669282 10.2147/DDDT.S357483 PMC9166517

[CR50] Szekeres R, Priksz D, Kiss R, Romanescu DD, Bombicz M, Varga B, Gesztelyi R, Szilagyi A, Takacs B, Tarjanyi V, Pelles-Tasko B, Forgacs I, Remenyik J, Szilvassy Z, Juhasz B (2023) Therapeutic aspects of *Prunus cerasus* extract in a rabbit model of atherosclerosis-associated diastolic dysfunction. Int J Mol Sci 24:13253. 10.3390/ijms241713253 10.3390/ijms241713253 PMC10488229 37686067

[CR51] Thummayot S, Tocharus C, Jumnongprakhon P, Suksamrarn A, Tocharus J (2018) Cyanidin attenuates Aβ(25–35)-induced neuroinflammation by suppressing NF-κB activity downstream of TLR4/NOX4 in human neuroblastoma cells. Acta Pharmacol Sin 39:1439–1452. 10.1038/aps.2017.203 29671417 10.1038/aps.2017.203 PMC6289386

[CR52] Verma S, Mazer CD, Connelly KA (2023) Inflammation and cholesterol at the crossroads of vascular risk. Cell Metabol 35:1095–1098. 10.1016/j.cmet.2023.06.011 10.1016/j.cmet.2023.06.011 37437543

[CR54] Wang L, Xu Z, Liao K, Zhao Y, Zhou L (2006) Study on ecology-biology of wild Cherry Plum in Xinjiang I. The analysis on ecology factors with characters of botanic, phynology, distribution. Xinjiang Agric Sci 43:87–95. 10.1016/S1872-2032(06)60050-4

[CR56] Wang Y, Chen X, Zhang Y, Chen X (2012a) Antioxidant activities and major anthocyanins of *Myrobalan* plum (*Prunus cerasifera* Ehrh). J Food Sci 77:C388-393. 10.1111/j.1750-3841.2012.02624.x 10.1111/j.1750-3841.2012.02624.x 22432436

[CR57] Wang Y, Zhang Y, Wang X, Liu Y, Xia M (2012b) Cyanidin-3-O-β-glucoside induces oxysterol efflux from endothelial cells: role of liver X receptor alpha. Atherosclerosis 223:299–305. 10.1016/j.atherosclerosis.2012.06.004 22749359 10.1016/j.atherosclerosis.2012.06.004

[CR53] Wang L, Huang Z, Huang W, Chen X, Shan P, Zhong P, Khan Z, Wang J, Fang Q, Liang G, Wang Y (2017) Inhibition of epidermal growth factor receptor attenuates atherosclerosis via decreasing inflammation and oxidative stress. Sci Rep 8:45917. 10.1038/srep45917 28374780 10.1038/srep45917 PMC5379239

[CR55] Wang P, Zeng G, Yan Y, Zhang SY, Dong Y, Zhang Y, Zhang X, Liu H, Zhang Z, Jiang C, Pang Y (2022) Disruption of adipocyte HIF-1α improves atherosclerosis through the inhibition of ceramide generation. Acta Pharm Sinica B 12:1899–1912. 10.1016/j.apsb.2021.10.001 10.1016/j.apsb.2021.10.001 PMC9279628 35847503

[CR58] Wolf D, Ley K (2019) Immunity and inflammation in atherosclerosis. Circul Res 124:315–327. 10.1161/circresaha.118.313591 10.1161/CIRCRESAHA.118.313591 PMC6342482 30653442

[CR59] Xin M, Xu A, Tian J, Wang L, He Y, Jiang H, Yang B, Li B, Sun Y (2024) Anthocyanins as natural bioactives with anti-hypertensive and atherosclerotic potential: health benefits and recent advances. Phytomedicine 132:155889. 10.1016/j.phymed.2024.155889 39047414 10.1016/j.phymed.2024.155889

[CR60] Yan F, Ding H, Sun Z, Liu J, Li J, Zhou D, Zhang W (2023) Glycoside combinations of Buyang Huanwu decoction ameliorate atherosclerosis via STAT3, HIF-1, and VEGF. Naunyn-Schmiedeberg’s archives of pharmacology 396:1187–1203. 10.1007/s00210-023-02389-6 10.1007/s00210-023-02389-6 36692827

[CR61] Yang S, Mi L, Wu J, Liao X, Xu Z (2023) Strategy for anthocyanins production: from efficient green extraction to novel microbial biosynthesis. Crit Rev Food Sci Nutr 63:9409–9424. 10.1080/10408398.2022.2067117 35486571 10.1080/10408398.2022.2067117

[CR62] Yao Y, Zhang X, Xu Y, Zhao Y, Song F, Tian Z, Zhao M, Liang Y, Ling W, Mao Y-H, Yang Y (2022) Cyanidin-3-O-β-glucoside attenuates platelet chemokines and their receptors in atherosclerotic inflammation of ApoE^–/–^ mice. J Agric Food Chem 70:8254–8263. 10.1021/acs.jafc.2c01844 35758304 10.1021/acs.jafc.2c01844

[CR63] Zaa CA, Marcelo Á, An J, Medina-Franco Z, J. L. and, Velasco-Velázquez MA (2023) Anthocyanins: molecular aspects on their neuroprotective activity. Biomolecules 13:1598. 10.3390/biom13111598 10.3390/biom13111598 PMC10669056 38002280

[CR64] Zeboudj L, Giraud A, Guyonnet L, Zhang Y, Laurans L, Esposito B, Vilar J, Chipont A, Papac-Milicevic N, Binder CJ, Tedgui A, Mallat Z, Tharaux PL, Ait-Oufella H (2018a) Selective EGFR (epidermal growth factor receptor) deletion in myeloid cells limits atherosclerosis-brief report. Arterioscler Thromb Vasc Biol 38:114–119. 10.1161/atvbaha.117.309927 29191921 10.1161/ATVBAHA.117.309927

[CR65] Zeboudj L, Maître M, Guyonnet L, Laurans L, Joffre J, Lemarie J, Bourcier S, Nour-Eldine W, Guérin C, Friard J, Wakkach A, Fabre E, Tedgui A, Mallat Z, Tharaux PL, Ait-Oufella H (2018b) Selective EGF-receptor inhibition in CD4^+^ T cells induces anergy and limits atherosclerosis. J Am Coll Cardiol 71:160–172. 10.1016/j.jacc.2017.10.084 29325640 10.1016/j.jacc.2017.10.084

[CR66] Zhai Y, Yang J, Zhang J, Yang J, Li Q, Zheng T (2021) Src-family protein tyrosine kinases: a promising target for treating cardiovascular diseases. Int J Med Sci 18:1216–1224. 10.7150/ijms.49241 33526983 10.7150/ijms.49241 PMC7847615

[CR67] Zhang P, Zhu H (2023) Anthocyanins in plant food: current status, genetic modification, and future perspectives. Molecules 28:866. 10.3390/molecules28020866 10.3390/molecules28020866 PMC9863750 36677927

[CR70] Zhao Y, Li Y, Liu Y, Yang YF (2015) Genetic diversity of wild *Prunus cerasifera* Ehrhart (wild cherry plum) in China revealed by simple-sequence repeat markers. Genet Mol Research: GMR 14:8407–8413. 10.4238/2015.July.28.7 26345767 10.4238/2015.July.28.7

[CR68] Zhao S, Tang X, Miao Z, Chen Y, Cao J, Song T, You D, Zhong Y, Lin Z, Wang D, Shi Z, Tang X, Wang D, Chen S, Wang L, Gu A, Chen F, Xie L, Huang Z, Wang H, Ji Y (2022) Hsp90 S-nitrosylation at Cys521, as a conformational switch, modulates cycling of Hsp90-AHA1-CDC37 chaperone machine to aggravate atherosclerosis. Redox Biol 52:102290. 10.1016/j.redox.2022.102290 35334246 10.1016/j.redox.2022.102290 PMC8942817

[CR69] Zhao W, Ouyang C, Zhang L, Wang J, Zhang J, Zhang Y, Huang C, Xiao Q, Jiang B, Lin F, Zhang C, Zhu M, Xie C, Huang X, Zhang B, Zhao W, He J, Chen S, Liu X, Lin D, Li Q, Wang Z (2024) The proto-oncogene tyrosine kinase c-SRC facilitates glioblastoma progression by remodeling fatty acid synthesis. Nat Commun 15:7455. 10.1038/s41467-024-51444-0 39198451 10.1038/s41467-024-51444-0 PMC11358276

