# Supplementary material for: Unraveling anti-atherosclerosis mechanism of anthocyanins from Xinjiang wild cherry plum (Prunus divaricata Ledeb) via network pharmacology and molecular docking
Source: Bioresour Bioprocess. 2025 Jun 6;12(1):53. doi: 10.1186/s40643-025-00900-w (PMC12144020; doi:10.1186/s40643-025-00900-w)
Supplement: Supplementary file 1 — Supplementary Material 1 [file 40643_2025_900_MOESM1_ESM.docx]

**Electronic Supplementary Material**

Table S1. The analysis results of core PPIs network by CytoHubba with MCC method.

Table S2. The docking score of anthocyanins and key hub targets (affinity, kcal/mol).

Table S3. The predicted hydrophobic interactions by PLIP.

Table S4. The predicted hydrogen bond interactions by PLIP.

Table S5. The predicted salt bridges by PLIP.

Table S6. The predicted π-stacking by PLIP.

Table S7. The predicted π-cation interaction by PLIP.

Figure S1. Three dimensional (3D) interaction of key targets with their optimal ligands.

**Table S1**. The analysis results of core PPIs network by CytoHubba with MCC method.

| **Node_name** | **Score** | **DMNC** | **MNC** | **Degree** | **EPC** | **Closeness** | **Betweenness** | **Stress** |
| --- | --- | --- | --- | --- | --- | --- | --- | --- |
| EGFR | 474 | 0.5122 | 12 | 12 | 6.805 | 12.5 | 19.45 | 62 |
| VEGFA | 438 | 0.5387 | 10 | 10 | 6.546 | 11.5 | 10.39 | 36 |
| IGF1R | 408 | 0.6415 | 8 | 8 | 6.022 | 10.5 | 2.42 | 12 |
| HIF1A | 384 | 0.6123 | 8 | 8 | 6.064 | 10.5 | 2.82 | 14 |
| HSP90AA1 | 378 | 0.5489 | 9 | 9 | 6.341 | 11 | 6.42 | 26 |
| CXCR4 | 180 | 0.5540 | 8 | 8 | 5.906 | 10.5 | 4.20 | 18 |
| SRC | 166 | 0.4773 | 9 | 9 | 6.26 | 11 | 9.03 | 32 |
| MTOR | 121 | 0.6482 | 5 | 6 | 5.133 | 9.5 | 3.23 | 10 |
| ITGB1 | 54 | 0.5230 | 6 | 6 | 5.316 | 9.5 | 1.95 | 8 |
| TNF | 44 | 0.4390 | 7 | 7 | 5.488 | 10 | 5.63 | 18 |
| TLR4 | 28 | 0.4025 | 7 | 7 | 5.599 | 10 | 5.82 | 20 |
| APP | 14 | 0.3890 | 5 | 5 | 4.838 | 9 | 2.23 | 8 |
| PTGS2 | 12 | 0.4737 | 4 | 4 | 4.494 | 8.5 | 0.33 | 2 |
| NFKB1 | 7 | 0.2842 | 4 | 5 | 4.302 | 9 | 4.07 | 14 |

**Note:** The targets were ranked in descending scores. EPC: Edge Percolated Component; DMNC: Maximum Neighborhood Component Centrality; MCC: Maximum Clique Centrality; MNC: Neighborhood Component Centrality.

**Table S2**. The docking score of anthocyanins and key hub targets (affinity, kcal/mol).

| **Target** | **PDB code** | **Cy3AcGlu** | | | **Cy3Glu** | | | **Cy3Xyl** | | | **Cy3Rut** | | | **Cy3Gal** | | | **Cy** | | |
| --- | --- | --- | --- | --- | --- | --- | --- | --- | --- | --- | --- | --- | --- | --- | --- | --- | --- | --- | --- |
| EGFR | 4zau | -8.44 | -8.44 | -8.43 | -8.32 | -8.32 | -8.28 | -- | -- | -- | -8.86 | -8.90 | -8.92 | -8.35 | -8.35 | -8.37 | -7.75 | -7.76 | -7.77 |
|  | 5gty | -9.26 | -9.10 | -8.78 | -9.02 | -9.76 | -8.78 | -- | -- | -- | -10.96 | -11.33 | -9.35 | -9.72 | -10.03 | -9.51 | -8.76 | -8.81 | -9.18 |
|  | 6duk | -8.42 | -8.08 | -8.07 | -8.71 | -8.50 | -8.49 | -- | -- | -- | -8.83 | -8.82 | -8.83 | -8.07 | -8.17 | -8.15 | -8.43 | -8.48 | -8.45 |
| VEGFA | 1mkg | -- | -- | -- | -7.76 | -7.50 | -7.43 | -- | -- | -- | -- | -- | -- | -7.90 | -7.86 | -7.86 | -- | -- | -- |
|  | 4kzn | -- | -- | -- | -6.38 | -6.36 | -6.39 | -- | -- | -- | -- | -- | -- | -6.22 | -6.09 | -6.16 | -- | -- | -- |
|  | 6zfl | -- | -- | -- | -7.61 | -7.64 | -7.63 | -- | -- | -- | -- | -- | -- | -7.07 | -7.34 | -7.05 | -- | -- | -- |
| HSP90AA1 | 2yi7 | -- | -- | -- | -8.65 | -8.92 | -7.98 | -- | -- | -- | -- | -- | -- | -8.61 | -8.60 | -8.66 | -- | -- | -- |
|  | 4bqg | -- | -- | -- | -10.56 | -10.54 | -7.91 | -- | -- | -- | -- | -- | -- | -9.86 | -9.83 | -7.74 | -- | -- | -- |
|  | 7lt0 | -- | -- | -- | -9.97 | -10.00 | -9.96 | -- | -- | -- | -- | -- | -- | -9.07 | -9.03 | -8.99 | -- | -- | -- |
| SRC | 1byg | -9.15 | -8.90 | -9.16 | -8.86 | -8.87 | -8.92 | -9.15 | -9.14 | -9.10 | -10.26 | -10.27 | -10.30 | -9.11 | -9.13 | -9.16 | -8.76 | -8.67 | -8.75 |
|  | 2bdf | -8.59 | -8.62 | -8.71 | -9.06 | -9.04 | -9.17 | -8.93 | -8.90 | -8.92 | -9.59 | -9.58 | -9.73 | -8.85 | -8.78 | -8.93 | -8.03 | -8.01 | -7.99 |
|  | 6ate | -8.40 | -8.46 | -8.38 | -9.18 | -9.20 | -9.19 | -9.43 | -9.46 | -9.44 | -8.84 | -9.11 | -7.71 | -9.09 | -8.67 | -9.11 | -9.70 | -9.67 | -9.68 |
| HIF1A | 4h6j | -6.12 | -6.15 | -6.05 | -6.34 | -6.32 | -6.31 | -6.65 | -6.09 | -6.10 | -6.43 | -6.39 | -6.43 | -6.11 | -6.10 | -6.11 | -- | -- | -- |
| CXCR4 | 2n55 | -- | -- | -- | -- | -- | -- | -- | -- | -- | -6.34 | -6.34 | -6.34 | -- | -- | -- | -5.66 | -5.65 | -5.45 |
|  | 3odu | -- | -- | -- | -- | -- | -- | -- | -- | -- | -9.50 | -9.81 | -9.81 | -- | -- | -- | -8.03 | -8.04 | -8.03 |
| IGF1R | 1m7n | -- | -- | -- | -- | -- | -- | -8.39 | -8.29 | -8.07 | -- | -- | -- | -- | -- | -- | -7.78 | -8.20 | -7.39 |
|  | 3o23 | -- | -- | -- | -- | -- | -- | -7.97 | -7.96 | -8.00 | -- | -- | -- | -- | -- | -- | -8.28 | -8.28 | -8.27 |
|  | 5fxr | -- | -- | -- | -- | -- | -- | -8.17 | -8.04 | -8.16 | -- | -- | -- | -- | -- | -- | -7.57 | -7.58 | -7.53 |
| AGPAT1 | 8erc | -8.06 | -7.40 | -7.96 | -8.16 | -7.97 | -7.11 | -7.32 | -7.19 | -8.15 | -9.17 | -9.18 | -9.20 | -7.97 | -8.31 | -8.29 | -7.76 | -8.49 | -8.64 |
| ENPP2 | 4zg7 | -9.15 | -9.10 | -8.04 | -8.70 | -8.30 | -8.30 | -8.69 | -8.69 | -9.08 | -9.51 | -9.35 | -9.40 | -8.59 | -8.50 | -9.01 | -8.34 | -8.17 | -8.29 |
|  | 5mhp | -9.34 | -9.40 | -8.10 | -7.87 | -8.98 | -8.98 | -9.32 | -9.21 | -9.20 | -10.35 | -10.29 | -10.40 | -8.93 | -8.92 | -7.67 | -8.37 | -8.44 | -8.46 |
|  | 8c3p | -8.56 | -8.68 | -9.21 | -8.78 | -9.09 | -8.79 | -9.56 | -9.53 | -9.56 | -9.95 | -9.89 | -9.54 | -9.08 | -9.03 | -9.05 | -8.87 | -8.86 | -8.88 |
| GPCPD1 | 2z0b | -8.74 | -8.27 | -8.04 | -8.29 | -8.32 | -8.30 | -8.56 | -9.16 | -8.72 | -9.34 | -9.34 | -8.84 | -8.63 | -8.86 | -8.53 | -8.16 | -8.12 | -7.52 |
| LCAT | 4xwg | -8.75 | -7.06 | -7.58 | -8.16 | -7.89 | -8.11 | -8.61 | -8.52 | -8.60 | -8.64 | -8.62 | -8.63 | -7.74 | -8.59 | -8.56 | -8.27 | -8.05 | -8.24 |
|  | 5txf | -7.84 | -8.03 | -8.03 | -7.66 | -7.65 | -7.93 | -7.78 | -7.87 | -7.87 | -7.85 | -9.08 | -9.02 | -7.64 | -7.72 | -7.69 | -8.01 | -8.06 | -7.92 |
|  | 6mvd | -7.85 | -7.66 | -7.14 | -7.89 | -8.27 | -8.28 | -7.55 | -7.54 | -7.55 | -8.55 | -8.82 | -8.78 | -8.19 | -7.63 | -8.28 | -8.25 | -8.20 | -7.73 |
| PLA2G1B | 3elo | -7.27 | -6.94 | -7.46 | -8.18 | -8.16 | -8.18 | -8.10 | -8.11 | -8.12 | -8.86 | -8.51 | -8.92 | -7.57 | -7.62 | -6.86 | -6.60 | -6.66 | -6.88 |
|  | 6q42 | -6.94 | -6.36 | -6.57 | -6.73 | -6.43 | -6.77 | -7.11 | -7.98 | -9.41 | -8.17 | -7.04 | -7.19 | -6.64 | -6.71 | -6.66 | -8.96 | -8.35 | -6.65 |
| PPARG | 2prg | -8.38 | -8.58 | -7.45 | -7.96 | -8.54 | -7.87 | -8.24 | -8.42 | -8.08 | -8.70 | -9.54 | -8.75 | -8.53 | -8.48 | -9.04 | -7.68 | -8.10 | -7.97 |
|  | 4ema | -8.60 | -8.61 | -8.61 | -8.35 | -8.38 | -8.38 | -8.31 | -8.30 | -8.27 | -10.00 | -9.81 | -9.87 | -8.44 | -8.45 | -8.42 | -7.83 | -7.42 | -7.47 |
|  | 5y2o | -8.29 | -8.73 | -8.41 | -8.62 | -8.09 | -7.77 | -7.92 | -8.94 | -8.74 | -9.16 | -9.26 | -9.19 | -8.59 | -8.28 | -8.31 | -8.39 | -7.99 | -8.15 |
|  | 5y2t | -8.81 | -8.66 | -8.77 | -7.84 | -7.84 | -8.77 | -8.80 | -8.00 | -8.78 | -8.70 | -8.76 | -8.57 | -8.98 | -7.85 | -8.65 | -8.07 | -8.16 | -8.49 |
|  | 6k0t | -7.85 | -8.76 | -8.15 | -8.84 | -8.81 | -8.82 | -8.35 | -8.52 | -8.53 | -9.12 | -9.34 | -9.44 | -8.01 | -8.22 | -8.25 | -8.12 | -8.02 | -8.02 |

--: The ligand has no corresponding target, thereby no interaction and docking score.

**Table S3. The predicted hydrophobic interactions by PLIP.**

| **Target-ligand complex** | **Index** | **Residue** | | **AA** | **Distance** | | **Ligand Atom** | | **Protein Atom** |
| --- | --- | --- | --- | --- | --- | --- | --- | --- | --- |
| ENPP2_5mhp_Cy3AcGlu | | | | | | | | | |
|  | 1 | 214A | | LEU | 3.61 | | 4 | | 1480 |
|  | 2 | 214A | | LEU | 3.49 | | 30 | | 1482 |
|  | 3 | 249A | | LYS | 3.38 | | 26 | | 1793 |
|  | 4 | 250A | | PHE | 3.53 | | 26 | | 1808 |
|  | 5 | 274A | | PHE | 3.75 | | 9 | | 2044 |
|  | 6 | 275A | | PHE | 3.42 | | 33 | | 2055 |
|  | 7 | 305A | | ALA | 4 | | 8 | | 2371 |
|  | 8 | 307A | | TYR | 3.98 | | 8 | | 2389 |
| HSP90AA1_4bqg_Cy3Glu | | |  | | |  | |  | |
|  | 1 | 107A | | LEU | 3.76 | | 5 | | 931 |
|  | 2 | 107A | | LEU | 3.68 | | 32 | | 932 |
|  | 3 | 107A | | LEU | 3.56 | | 8 | | 929 |
|  | 4 | 138A | | PHE | 3.73 | | 28 | | 1187 |
|  | 5 | 162A | | TRP | 3.94 | | 9 | | 1434 |
| ENPP2_8c3p_Cy3Xyl | | |  | | |  | |  | |
|  | 1 | 214B | | LEU | 3.35 | | 9 | | 9174 |
|  | 2 | 215B | | TYR | 3.72 | | 8 | | 9185 |
|  | 3 | 250B | | PHE | 3.8 | | 28 | | 9512 |
|  | 4 | 255B | | TRP | 3.5 | | 25 | | 9586 |
|  | 5 | 261B | | TRP | 3.42 | | 8 | | 9642 |
|  | 6 | 275B | | PHE | 3.97 | | 8 | | 9769 |
| EGFR_5gty_Cy3Rut | | |  | | |  | |  | |
|  | 1 | 910C | | PHE | 3.68 | | 23 | | 7606 |
|  | 2 | 937C | | PRO | 3.68 | | 19 | | 7850 |
|  | 3 | 948E | | VAL | 3.85 | | 13 | | 13504 |
|  | 4 | 948E | | VAL | 3.85 | | 19 | | 13505 |
|  | 5 | 970A | | LYS | 3.69 | | 40 | | 2516 |
|  | 6 | 982C | | GLN | 3.94 | | 16 | | 8299 |
| EGFR_5gty_Cy3Gal | | |  | | |  | |  | |
|  | 1 | 910C | | PHE | 3.69 | | 4 | | 7596 |
|  | 2 | 910C | | PHE | 3.89 | | 5 | | 7594 |
|  | 3 | 932E | | ARG | 3.61 | | 31 | | 13346 |
|  | 4 | 936C | | PRO | 3.61 | | 9 | | 7830 |
| SRC_6ate_Cyanidin | | |  | | |  | |  | |
|  | 1 | 276A | | LEU | 3.81 | | 10 | | 206 |
|  | 2 | 284A | | VAL | 3.96 | | 8 | | 269 |
|  | 3 | 296A | | ALA | 3.75 | | 6 | | 391 |
|  | 4 | 298A | | LYS | 3.63 | | 15 | | 406 |
|  | 5 | 341A | | THR | 3.81 | | 15 | | 759 |
|  | 6 | 396A | | LEU | 3.9 | | 2 | | 1298 |
| ENPP2_5mhp_Cy3Rut | | |  | | |  | |  | |
|  | 1 | 214A | | LEU | 3.88 | | 14 | | 1490 |
|  | 2 | 214A | | LEU | 3.81 | | 19 | | 1492 |
|  | 3 | 274A | | PHE | 3.86 | | 16 | | 2054 |
|  | 4 | 275A | | PHE | 3.94 | | 40 | | 2066 |
|  | 5 | 275A | | PHE | 3.49 | | 22 | | 2065 |
| SRC_1byg_Cy3Rut | | |  | | |  | |  | |
|  | 1 | 209A | | VAL | 3.86 | | 14 | | 265 |
|  | 2 | 209A | | VAL | 3.75 | | 12 | | 264 |
|  | 3 | 220A | | ALA | 3.41 | | 13 | | 371 |
|  | 4 | 222A | | LYS | 3.96 | | 23 | | 387 |
|  | 5 | 249A | | VAL | 3.61 | | 19 | | 630 |
|  | 6 | 264A | | ILE | 3.55 | | 22 | | 749 |
|  | 7 | 266A | | THR | 3.45 | | 23 | | 765 |
|  | 8 | 333A | | PHE | 3.32 | | 22 | | 1403 |
| PPARG_4ema_Cy3Rut | | |  | | |  | |  | |
|  | 1 | 323B | | HIS | 3.8 | | 16 | | 3494 |
|  | 2 | 373A | | LYS | 3.92 | | 40 | | 1523 |
| GPCPD1_2z0b_Cy3Rut | | |  | | |  | |  | |
|  | 1 | 44B | | ALA | 3.54 | | 22 | | 1439 |
|  | 2 | 78B | | PHE | 3.79 | | 13 | | 1735 |
|  | 3 | 80C | | GLU | 3.78 | | 19 | | 2802 |
|  | 4 | 81B | | PRO | 3.36 | | 40 | | 1765 |
| AGPAT1_8erc_Cy3Rut | | |  | | |  | |  | |
|  | 1 | 245A | | PHE | 3.75 | | 40 | | 2367 |
|  | 2 | 245A | | PHE | 3.49 | | 13 | | 2369 |
|  | 3 | 245A | | PHE | 3.95 | | 19 | | 2371 |
|  | 4 | 248A | | ALA | 3.79 | | 40 | | 2399 |
|  | 5 | 355A | | TRP | 3.85 | | 16 | | 3426 |
|  | 6 | 362A | | TYR | 3.73 | | 23 | | 3489 |
|  | 7 | 414A | | PHE | 3.54 | | 23 | | 4007 |
|  | 8 | 417A | | LEU | 3.1 | | 22 | | 4034 |
| LCAT_5txf_Cy3Rut | | |  | | |  | |  | |
|  | 1 | 366D | | PRO | 3.99 | | 40 | | 14462 |
|  | 2 | 369C | | LEU | 3.6 | | 12 | | 10814 |
|  | 3 | 369C | | LEU | 3.36 | | 19 | | 10811 |
| IGF1R_1m7n_Cy3Xyl | | |  | | |  | |  | |
|  | 1 | 1047B | | GLU | 3.74 | | 9 | | 3597 |
|  | 2 | 1050B | | VAL | 3.68 | | 8 | | 3622 |
|  | 3 | 1123B | | LEU | 3.47 | | 28 | | 4252 |
| HIF1A_4h6j_Cy3Xyl | | |  | | |  | |  | |
|  | 1 | 276A | | TYR | 3.81 | | 28 | | 419 |
|  | 2 | 291A | | HIS | 3.89 | | 8 | | 578 |
|  | 3 | 295A | | PHE | 3.65 | | 9 | | 627 |
| VEGFA_1mkg_Cy3Gal | | |  | | |  | |  | |
|  | 1 | 46C | | ILE | 3.54 | | 8 | | 2219 |
|  | 2 | 66B | | LEU | 3.67 | | 8 | | 1470 |
|  | 3 | 66B | | LEU | 3.84 | | 5 | | 1471 |
|  | 4 | 83C | | ILE | 3.96 | | 31 | | 2554 |

**Table S4. The predicted hydrogen bond interactions by PLIP.**

| **Target-ligand complex** | **Index** | **Residue** | **AA** | **Distance H-A** | **Distance D-A** | **Donor Angle** | **Protein donor?** | **Side chain** | **Donor Atom** | **Acceptor Atom** |
| --- | --- | --- | --- | --- | --- | --- | --- | --- | --- | --- |
| HSP90AA1_4bqg_Cy3Glu | | |  |  |  |  |  |  |  |  |
|  | 1 | 51A | ASN | 3.35 | 3.82 | 112.01 | ⨯ | ⨯ | 23 [O3] | 392 [O2] |
|  | 2 | 51A | ASN | 2.54 | 3.38 | 140 | √ | √ | 395 [Nam] | 25 [O3] |
|  | 3 | 97A | GLY | 2.56 | 3.16 | 117.56 | √ | ⨯ | 834 [Nam] | 19 [O3] |
|  | 4 | 137A | GLY | 2.6 | 3.26 | 121.65 | √ | ⨯ | 1178 [Nam] | 33 [O3] |
|  | 5 | 138A | PHE | 3.02 | 3.92 | 147.58 | √ | ⨯ | 1183 [Nam] | 33 [O3] |
|  | 6 | 162A | TRP | 2.72 | 3.12 | 103.76 | √ | √ | 1433 [Nar] | 39 [O2] |
|  | 7 | 184A | THR | 2.28 | 2.92 | 122.09 | ⨯ | √ | 21 [O3] | 1626 [O3] |
|  | 8 | 184A | THR | 2.86 | 3.29 | 107.67 | ⨯ | √ | 19 [O3] | 1626 [O3] |
| ENPP2_8c3p_Cy3Xyl | | |  |  |  |  |  |  |  |  |
|  | 1 | 82B | SER | 2.11 | 2.94 | 145.49 | √ | √ | 7921 [O3] | 18 [O3] |
|  | 2 | 83B | TYR | 2.73 | 3.13 | 106.08 | √ | √ | 7935 [O3] | 32 [O3] |
|  | 3 | 214B | LEU | 2.62 | 3.09 | 110.21 | ⨯ | ⨯ | 36 [O2] | 9173 [O2] |
|  | 4 | 249B | LYS | 2.12 | 3.03 | 155.24 | ⨯ | ⨯ | 30 [O3] | 9496 [O2] |
|  | 5 | 250B | PHE | 2.23 | 2.98 | 133.32 | ⨯ | ⨯ | 32 [O3] | 9509 [O2] |
|  | 6 | 276B | TRP | 2.2 | 3 | 138.33 | ⨯ | ⨯ | 18 [O3] | 9776 [O2] |
| EGFR_5gty_Cy3Rut | | |  |  |  |  |  |  |  |  |
|  | 1 | 909C | THR | 2.96 | 3.79 | 143.41 | ⨯ | ⨯ | 26 [O3] | 7593 [O2] |
|  | 2 | 912C | SER | 3.31 | 4.08 | 140.31 | √ | √ | 7621 [O3] | 26 [O3] |
|  | 3 | 942A | ASP | 2.22 | 2.74 | 112.61 | ⨯ | √ | 45 [O3] | 2234 [O-] |
|  | 4 | 944E | TYR | 2.56 | 3.17 | 122.73 | √ | √ | 13469 [O3] | 51 [O3] |
|  | 5 | 944E | TYR | 2.69 | 3.17 | 111.25 | ⨯ | √ | 51 [O3] | 13469 [O3] |
|  | 6 | 977A | ARG | 3.06 | 3.43 | 102.74 | √ | √ | 2594 [Ng+] | 47 [O3] |
|  | 7 | 977A | ARG | 3.51 | 3.83 | 100.99 | √ | √ | 2595 [Ng+] | 47 [O3] |
|  | 8 | 977A | ARG | 2.7 | 3.43 | 132.73 | ⨯ | √ | 47 [O3] | 2594 [Ng+] |
| EGFR_5gty_Cy3Gal | | |  |  |  |  |  |  |  |  |
|  | 1 | 806C | LYS | 2.75 | 3.58 | 137.72 | √ | √ | 6698 [N3+] | 33 [O3] |
|  | 2 | 909C | THR | 2.88 | 3.46 | 119.03 | ⨯ | ⨯ | 39 [O2] | 7581 [O2] |
|  | 3 | 912C | SER | 2.25 | 3.04 | 140.32 | √ | √ | 7609 [O3] | 39 [O2] |
|  | 4 | 932E | ARG | 2.04 | 3.03 | 162.17 | √ | √ | 13349 [Ng+] | 23 [O3] |
|  | 5 | 932E | ARG | 2.49 | 3.35 | 140.85 | √ | √ | 13352 [Ng+] | 23 [O3] |
|  | 6 | 932E | ARG | 1.93 | 2.8 | 141.55 | √ | ⨯ | 13342 [Nam] | 35 [O3] |
|  | 7 | 938C | ILE | 2.13 | 3.15 | 178.17 | √ | ⨯ | 7840 [Nam] | 37 [O2] |
|  | 8 | 982C | GLN | 2.31 | 2.89 | 117.54 | ⨯ | √ | 23 [O3] | 8291 [O2] |
|  | 9 | 982C | GLN | 2.48 | 3.12 | 119.75 | √ | ⨯ | 8283 [Nam] | 19 [O3] |
| SRC_6ate_Cyanidin | | |  |  |  |  |  |  |  |  |
|  | 1 | 298A | LYS | 2.97 | 3.31 | 100.57 | √ | √ | 410 [N3+] | 19 [O3] |
|  | 2 | 341A | THR | 2.35 | 2.77 | 105.89 | √ | √ | 760 [O3] | 21 [O2] |
|  | 3 | 342A | GLU | 2.44 | 2.92 | 110.24 | ⨯ | ⨯ | 21 [O2] | 766 [O2] |
|  | 4 | 344A | MET | 3.11 | 3.93 | 138.17 | √ | ⨯ | 787 [Nam] | 23 [O2] |
|  | 5 | 407A | ASP | 2.25 | 3.26 | 169.9 | √ | ⨯ | 1385 [Nam] | 17 [O3] |
| ENPP2_5mhp_Cy3Rut | | |  |  |  |  |  |  |  |  |
|  | 1 | 170A | SER | 3.27 | 3.66 | 107.05 | √ | √ | 1072 [O3] | 30 [O2] |
|  | 2 | 214A | LEU | 2.38 | 3.09 | 129.92 | ⨯ | ⨯ | 28 [O2] | 1489 [O2] |
|  | 3 | 273A | THR | 2.09 | 3 | 155.23 | ⨯ | √ | 43 [O3] | 2044 [O3] |
|  | 4 | 274A | PHE | 2.15 | 3.13 | 159.71 | √ | ⨯ | 2047 [Nam] | 45 [ |
| SRC_1byg_Cy3Rut | |  |  |  |  |  |  |  |  |  |
|  | 1 | 203A | LYS | 2.92 | 3.76 | 140.04 | √ | ⨯ | 205 [Nam] | 37 [O3] |
|  | 2 | 249A | VAL | 2.5 | 3.23 | 132.2 | ⨯ | ⨯ | 24 [O3] | 628 [O2] |
|  | 3 | 267A | GLU | 3.72 | 4.02 | 101.13 | ⨯ | ⨯ | 30 [O2] | 772 [O2] |
|  | 4 | 269A | MET | 1.93 | 2.92 | 164.27 | √ | ⨯ | 793 [Nam] | 30 [O2] |
|  | 5 | 319A | ASN | 3.24 | 3.78 | 116.24 | ⨯ | √ | 45 [O3] | 1280 [O2] |
|  | 6 | 331A | SER | 2.21 | 3.02 | 141.43 | √ | √ | 1387 [O3] | 32 [O3] |
|  | 7 | 332A | ASP | 2.37 | 3.38 | 171.15 | √ | ⨯ | 1390 [Nam] | 24 [O3] |
|  | 8 | 333A | PHE | 3.31 | 4.06 | 131.34 | √ | ⨯ | 1399 [Nam] | 24 [O3] |
|  | 9 | 333A | PHE | 3.45 | 3.95 | 114.56 | ⨯ | ⨯ | 32 [O3] | 1402 [O2] |
| PPARG_4ema_Cy3Rut | | |  |  |  |  |  |  |  |  |
|  | 1 | 320B | TYR | 1.81 | 2.71 | 151.5 | ⨯ | ⨯ | 28 [O2] | 3466 [O2] |
|  | 2 | 366A | PRO | 2.72 | 3.13 | 106.15 | ⨯ | ⨯ | 43 [O3] | 1452 [O2] |
|  | 3 | 397B | ARG | 1.98 | 2.79 | 134.68 | √ | √ | 4201 [Ng+] | 32 [O3] |
|  | 4 | 397B | ARG | 2.29 | 3.18 | 145.85 | √ | √ | 4198 [Ng+] | 49 [O3] |
|  | 5 | 443B | ARG | 2.28 | 3.23 | 163.74 | √ | √ | 4650 [Ng+] | 49 [O3] |
|  | 6 | 443B | ARG | 3.41 | 3.75 | 102.12 | √ | √ | 4651 [Ng+] | 49 [O3] |
|  | 7 | 443B | ARG | 2.36 | 3.23 | 148.81 | ⨯ | √ | 49 [O3] | 4650 [Ng+] |
|  | 8 | 444A | GLN | 1.9 | 2.84 | 151.59 | √ | √ | 2204 [Nam] | 1 [O3] |
| CXCR4_3odu_Cy3Rut | | |  |  |  |  |  |  |  |  |
|  | 1 | 32A | GLU | 3.07 | 3.48 | 107.49 | ⨯ | √ | 51 [O3] | 116 [O-] |
|  | 2 | 97A | ASP | 2.15 | 3.06 | 155.49 | ⨯ | √ | 45 [O3] | 750 [O.co2] |
|  | 3 | 183A | ARG | 2.61 | 3.21 | 117.66 | √ | √ | 1589 [Ng+] | 45 [O3] |
|  | 4 | 183A | ARG | 2.61 | 3.21 | 117.4 | √ | √ | 1588 [Ng+] | 45 [O3] |
|  | 5 | 186A | CYS | 3.13 | 4.09 | 173.74 | ⨯ | ⨯ | 28 [O2] | 1622 [O2] |
|  | 6 | 187A | ASP | 2.24 | 3.18 | 161.96 | ⨯ | √ | 32 [O3] | 1633 [O.co2] |
|  | 7 | 200A | GLN | 3.41 | 4.05 | 122.95 | √ | √ | 1773 [Nam] | 26 [O3] |
|  | 8 | 200A | GLN | 2.27 | 2.91 | 122.33 | ⨯ | √ | 26 [O3] | 1774 [O2] |
|  | 9 | 255A | TYR | 2.02 | 2.94 | 162.27 | √ | √ | 2315 [O3] | 24 [O3] |
| GPCPD1_2z0b_Cy3Rut | | |  |  |  |  |  |  |  |  |
|  | 1 | 45B | LEU | 2.7 | 3.62 | 157.46 | ⨯ | ⨯ | 26 [O3] | 1444 [O2] |
|  | 2 | 80C | GLU | 3.15 | 3.74 | 120.21 | ⨯ | ⨯ | 24 [O3] | 2801 [O2] |
|  | 3 | 88A | CYS | 3.16 | 3.93 | 133.43 | √ | ⨯ | 780 [N3+] | 51 [O3] |
|  | 4 | 88A | CYS | 2.07 | 2.9 | 142.35 | ⨯ | ⨯ | 51 [O3] | 783 [O2] |
|  | 5 | 89A | GLN | 3.28 | 4.01 | 134.36 | ⨯ | √ | 28 [O2] | 797 [O2] |
| AGPAT1_8erc_Cy3Rut | | |  |  |  |  |  |  |  |  |
|  | 1 | 8A | TYR | 2.42 | 3.14 | 130.6 | ⨯ | √ | 47 [O3] | 135 [O3] |
|  | 2 | 102A | ASN | 2.11 | 3.02 | 147.78 | √ | √ | 979 [Nam] | 49 [O3] |
|  | 3 | 244A | ARG | 2.01 | 2.8 | 132.66 | √ | √ | 2354 [Ng+] | 24 [O3] |
|  | 4 | 249A | TRP | 2.21 | 3.02 | 135.64 | √ | √ | 2411 [Nar] | 43 [O3] |
|  | 5 | 321A | ASN | 2.53 | 3.13 | 117.08 | √ | √ | 3067 [Nam] | 30 [O2] |
|  | 6 | 356A | HIS | 3.04 | 3.92 | 145.82 | √ | √ | 3438 [Npl] | 30 [O2] |
| LCAT_5txf_Cy3Rut | |  |  |  |  |  |  |  |  |  |
|  | 1 | 357C | GLY | 2.05 | 2.9 | 144 | ⨯ | ⨯ | 30 [O2] | 10687 [O2] |
|  | 2 | 360C | GLN | 2.33 | 3.08 | 128.55 | √ | √ | 10721 [Nam] | 10 [O2] |
|  | 3 | 360D | GLN | 2.6 | 3.34 | 133.59 | ⨯ | √ | 28 [O2] | 14401 [O2] |
|  | 4 | 367D | VAL | 1.97 | 2.98 | 170.49 | √ | ⨯ | 14465 [Nam] | 43 [O3] |
|  | 5 | 369C | LEU | 2.32 | 3.09 | 131.03 | √ | ⨯ | 10807 [Nam] | 24 [O3] |
|  | 6 | 369C | LEU | 1.95 | 2.85 | 152.63 | ⨯ | ⨯ | 24 [O3] | 10810 [O2] |
|  | 7 | 369D | LEU | 3.06 | 3.85 | 139.42 | ⨯ | ⨯ | 32 [O3] | 14489 [O2] |
| PLA2G1B_6q42_Cy3Xyl | | |  |  |  |  |  |  |  |  |
|  | 1 | 23A | ASN | 2.36 | 3.28 | 156.48 | ⨯ | √ | 18 [O3] | 267 [O2] |
|  | 2 | 24A | ASN | 2.17 | 3 | 142.61 | ⨯ | √ | 30 [O3] | 278 [O2] |
|  | 3 | 24B | ASN | 2.76 | 3.55 | 139.2 | ⨯ | √ | 22 [O3] | 1495 [O2] |
|  | 4 | 25A | TYR | 2.34 | 3.17 | 142.97 | ⨯ | ⨯ | 32 [O3] | 285 [O2] |
|  | 5 | 26B | GLY | 2.46 | 2.8 | 100.32 | ⨯ | ⨯ | 34 [O2] | 1516 [O2] |
|  | 6 | 30B | GLY | 2.73 | 3.67 | 161.43 | ⨯ | ⨯ | 36 [O2] | 1549 [O2] |
|  | 7 | 31A | LEU | 2.5 | 3.38 | 143.76 | √ | ⨯ | 334 [Nam] | 13 [O3] |
|  | 8 | 119B | ASP | 2.49 | 3.2 | 132.96 | √ | √ | 2400 [O3] | 18 [O3] |
|  | 9 | 120B | THR | 2.88 | 3.77 | 146.71 | √ | ⨯ | 2403 [Nam] | 20 [O3] |
|  | 10 | 120B | THR | 3.19 | 3.55 | 104.14 | √ | √ | 2409 [O3] | 13 [O3] |
|  | 11 | 121B | LYS | 2.41 | 3.41 | 164.76 | √ | ⨯ | 2412 [Nam] | 18 [O3] |
| IGF1R_1m7n_Cy3Xyl | | |  |  |  |  |  |  |  |  |
|  | 1 | 1047B | GLU | 3.24 | 3.6 | 104.2 | ⨯ | √ | 34 [O2] | 3599 [O.co2] |
|  | 2 | 1132B | ASP | 3.41 | 4.04 | 124.41 | ⨯ | √ | 18 [O3] | 4353 [O.co2] |
|  | 3 | 1137B | ASN | 2.87 | 3.83 | 167.95 | ⨯ | √ | 20 [O3] | 4400 [O2] |
| HIF1A_4h6j_Cy3Xyl | | |  |  |  |  |  |  |  |  |
|  | 1 | 248A | LEU | 2.7 | 3.09 | 104.85 | ⨯ | ⨯ | 34 [O2] | 142 [O2] |
|  | 2 | 274A | SER | 2.33 | 2.8 | 109.89 | √ | √ | 403 [O3] | 22 [O3] |
|  | 3 | 288A | THR | 2.35 | 3.03 | 126.07 | ⨯ | √ | 30 [O3] | 549 [O3] |
| VEGFA_1mkg_Cy3Gal | | |  |  |  |  |  |  |  |  |
|  | 1 | 25B | TYR | 2.07 | 2.95 | 154.42 | √ | √ | 1093 [O3] | 33 [O3] |
|  | 2 | 84C | LYS | 3.07 | 3.95 | 150.46 | ⨯ | ⨯ | 35 [O3] | 2560 [O2] |
|  | 3 | 86C | HIS | 3.41 | 3.88 | 109.64 | √ | ⨯ | 2577 [Nam] | 35 [O3] |
|  | 4 | 87C | GLN | 2.31 | 3.23 | 149 | √ | ⨯ | 2590 [Nam] | 35 [O3] |
|  | 5 | 88C | GLY | 2.14 | 3.14 | 166.3 | √ | ⨯ | 2602 [Nam] | 35 [O3] |
|  | 6 | 103B | GLU | 2.32 | 2.75 | 106.09 | ⨯ | √ | 25 [O3] | 1836 [O.co2] |
|  | 7 | 104B | CYS | 2.44 | 3.39 | 154.84 | √ | ⨯ | 1838 [Nam] | 33 [O3] |
|  | 8 | 105B | ARG | 2.5 | 3.11 | 119.69 | √ | √ | 1854 [Ng+] | 19 [O3] |
|  | 9 | 105B | ARG | 2.59 | 3.18 | 116.63 | √ | √ | 1852 [Ng+] | 25 [O3] |
|  | 10 | 105B | ARG | 2.51 | 3.1 | 116.68 | √ | √ | 1855 [Ng+] | 25 [O3] |

**Table S5. The predicted salt bridges by PLIP.**

| **Target-ligand complex** | **Index** | **Residue** | **AA** | **Distance** | **Protein positive?** | **Ligand Group** | **Ligand Atoms** |
| --- | --- | --- | --- | --- | --- | --- | --- |
| EGFR_5gty_Cy3Rut | | | | | | | |
|  | 1 | 806C | LYS | 3.82 | √ | Carboxylate | 35, 37 |
|  | 2 | 806C | LYS | 3.32 | √ | Carboxylate | 1, 7 |
| SRC_1byg_Cy3Rut | | | | | | | |
|  | 1 | 222A | LYS | 3.68 | √ | Carboxylate | 1, 7 |
| PPARG_4ema_Cy3Rut | | | | | | | |
|  | 1 | 443B | ARG | 4.71 | √ | Carboxylate | 1, 7 |
| IGF1R_1m7n_Cy3Xyl | | | | | | | |
|  | 1 | 1130B | HIS | 3.9 | √ | Carboxylate | 11, 13 |
| VEGFA_1mkg_Cy3Gal | | | | | | | |
|  | 1 | 105B | ARG | 4.46 | √ | Carboxylate | 11, 13 |

**Table S6. The predicted π-stacking by PLIP.**

| Target-ligand complex | Index | Residue | AA | Distance | Angle | Offset | Stacking  Type | Ligand Atoms |
| --- | --- | --- | --- | --- | --- | --- | --- | --- |
| ENPP2_5mhp_Cy3AcGlu | | | | | | | | |
|  | 1 | 307A | TYR | 3.81 | 5.33 | 1.27 | P | 29, 30, 31, 32, 33, 34 |
| HSP90AA1_4bqg_Cy3Glu | | | | | | | | |
|  | 1 | 138A | PHE | 4.3 | 2.97 | 1.91 | P | 1, 2, 3, 4, 5, 6 |
|  | 2 | 138A | PHE | 4.11 | 2.98 | 1.56 | P | 5, 6, 7, 8, 9, 10 |
|  | 3 | 162A | TRP | 5.1 | 78.72 | 1.79 | T | 5, 6, 7, 8, 9, 10 |
| ENPP2_5mhp_Cy3Rut | | | | | | | | |
|  | 1 | 307A | TYR | 3.85 | 11.43 | 1.09 | P | 18, 19, 20, 21, 22, 23 |
| PPARG_4ema_Cy3Rut | | | | | | | | |
|  | 1 | 320B | TYR | 5.23 | 60.77 | 1.9 | T | 11, 12, 13, 15, 16, 17 |
|  | 2 | 320B | TYR | 4.92 | 61.14 | 0.6 | T | 8, 9, 10, 11, 12, 14 |
| CXCR4_3odu_Cy3Rut | | | | | | | | |
|  | 1 | 94A | TRP | 4.18 | 26.7 | 0.6 | P | 11, 12, 13, 15, 16, 17 |
|  | 2 | 113A | HIS | 4.08 | 3.26 | 1.65 | P | 11, 12, 13, 15, 16, 17 |
|  | 3 | 116A | TYR | 5.44 | 69.82 | 1.6 | T | 11, 12, 13, 15, 16, 17 |
| AGPAT1_8erc_Cy3Rut | | | | | | | | |
|  | 1 | 356A | HIS | 4.74 | 74.28 | 1.01 | T | 11, 12, 13, 15, 16, 17 |
| HIF1A_4h6j_Cy3Xyl | | | | | | | | |
|  | 1 | 276A | TYR | 3.86 | 6.94 | 1.01 | P | 24, 25, 26, 27, 28, 29 |

**Table S7. The predicted π-cation interaction by PLIP.**

| Target-ligand complex | Index | Residue | AA | Distance | Offset | Protein charged | Ligand  Group | Ligand Atoms |
| --- | --- | --- | --- | --- | --- | --- | --- | --- |
| EGFR_5gty_Cy3Gal | | | | | | | | |
|  | 1 | 806C | LYS | 3.65 | 1.55 | √ | Aromatic | 27, 28, 29, 30, 31, 32 |


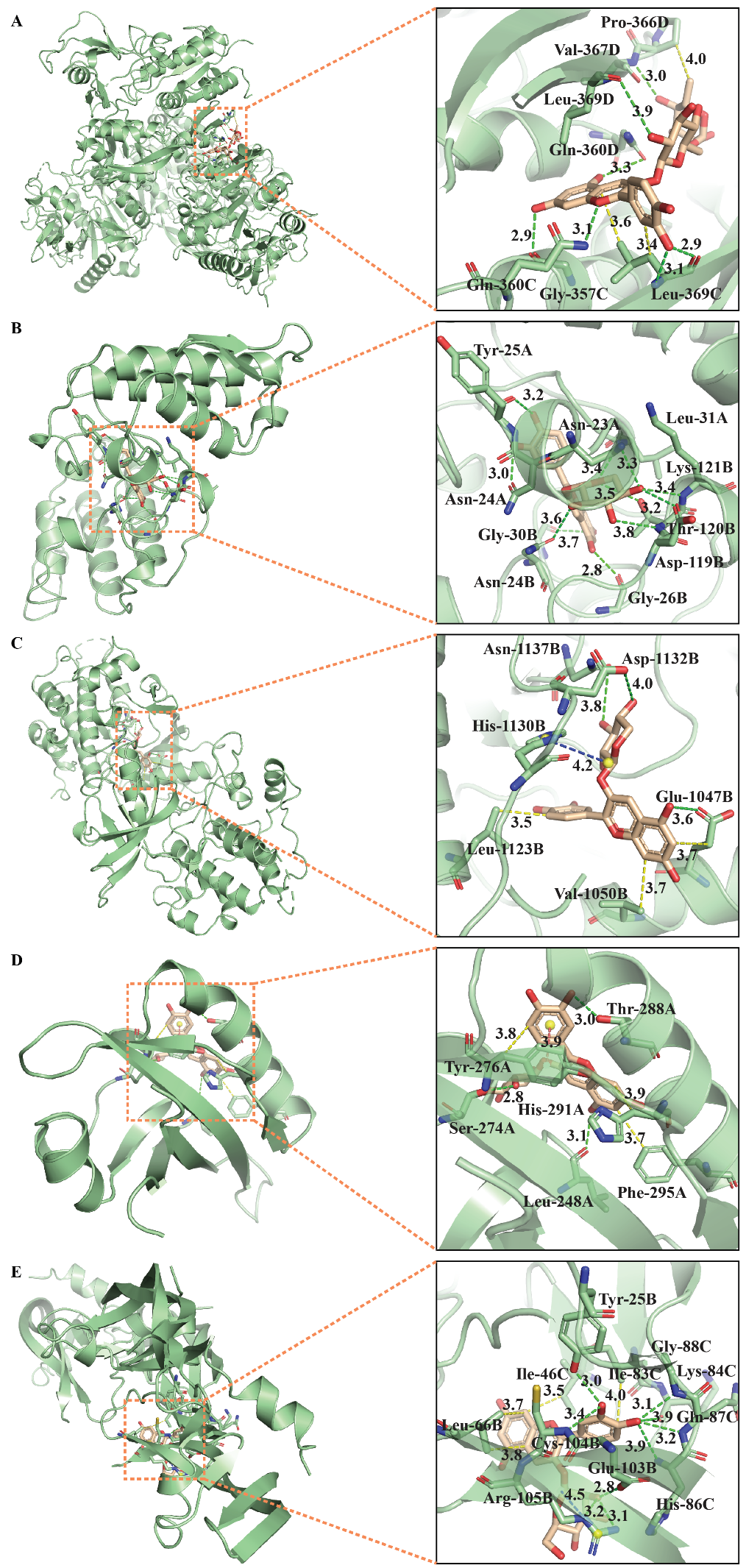


**Figure S1.** **Molecular interaction and docking poses of key targets with their optimal ligands.** (A) LCAT(5txf)-Cy3Rut, (B) PLA2G1B(6q42)-Cy3Xyl, (C) IGF1R(1m7n)-Cy3Xyl, (D) HIF1A(4h6j)-Cy3Xyl and (E) VEGFA(1mkg)-Cy3Gal. Dotted lines with yellow, purplish red and green colors represented hydrophobic, hydrogen bond and π-stacking (π-cation) interactions, respectively.
